# Analysis of coding variants in the human *FTO* gene from the gnomAD database

**DOI:** 10.1371/journal.pone.0248610

**Published:** 2022-01-06

**Authors:** Mauro Lúcio Ferreira Souza Junior, Jaime Viana de Sousa, João Farias Guerreiro

**Affiliations:** 1 Laboratory of Human and Medical Genetics, Institute of Biological Sciences, Federal University of Pará, Belém, PA, Brazil; 2 Federal Rural University of Amazon, Capanema Campus, PA, Brazil; Universitat Pompeu Fabra, SPAIN

## Abstract

Single nucleotide polymorphisms (SNPs) in the first intron of the *FTO* gene reported in 2007 continue to be the known variants with the greatest effect on adiposity in different human populations. Coding variants in the *FTO* gene, on the other hand, have been little explored, although data from complete sequencing of the exomes of various populations are available in public databases and provide an excellent opportunity to investigate potential functional variants in *FTO*. In this context, this study aimed to track nonsynonymous variants in the exons of the *FTO* gene in different population groups employing the gnomAD database and analyze the potential functional impact of these variants on the *FTO* protein using five publicly available pathogenicity prediction programs. The findings revealed 345 rare mutations, of which 321 are missense (93%), 19 are stop gained (5.6%) and five mutations are located in the splice region (1.4%). Of these, 134 (38.8%) were classified as pathogenic, 144 (41.7%) as benign and 67 (19.5%) as unknown. The available data, however, suggest that these variants are probably not associated with BMI and obesity, but instead, with other diseases. Functional studies are, therefore, required to identify the role of these variants in disease genesis.

## Introduction

The Fat mass and obesity-associated gene, also known as *FTO* (alpha-ketoglutarate-dependent dioxygenase), was the first obesity susceptibility gene identified through Genome-Wide Association Studies (GWAS) and remains the locus with the greatest effect on adiposity in different human populations. Four independent GWAS published in 2007 reported a significant association between body mass index and body fat and common genetic *FTO* gene variants, specifically, a group of single nucleotide polymorphisms (SNPs) in the first intron of this gene. The *FTO* was identified for the first time in Europeans in 2007 [1], and shortly thereafter, its association with BMI and obesity risk was confirmed by three other studies [2–4]. This association has been replicated in other populations (Asians, Hispanics and Native Americans), although conflicting results have been observed in African/African-American populations [5, 6]. The frequencies of risk alleles vary substantially between different ethnic groups, which may explain, to some degree, the differences in estimates concerning the effects of these alleles on the BMI. Different populations are characterized by several specific patterns of tightly linked SNP haplotypes associated with the phenotype [7].

The *FTO* gene, located on chromosome 16q12.2, is expressed in a wide range of tissues, as it is a maintenance gene that maintains the CpG islands in gene promoters [6]. It contains nine exons and spans approximately 410 kb, unusually large for a maintenance gene. It encodes a 2-oxoglutarate-dependent oxygenase that performs oxidative RNA/DNA demethylation, and available data suggest that *FTO* plays a role in the arcuate nuclei of the hypothalamus, where it mediates energy balance and eating behavior [7]. The intronic location of common SNPs associated with BMI and obesity within a 47 kb region that covers parts of the first two introns and exon 2 of *FTO* [1] indicates that the amino acid sequence of *FTO* protein does not exert its effects through functional mutations, and is more likely to play a role in transcription regulation through its effect on the expression of *FTO* gene and/or neighboring genes, such as the IRX3/IRX5 genes, specifically in adipocytes. Experimental data [8] have confirmed that *FTO* intron 1 is involved in enhancer activation, as previously described by another study [9], and regulates the expression of the *IRX3* and *IRX5* loci, which are vital for adipocyte maturation [7, 6].

Data obtained in 2017 from the NHGRI-EBI GWAS catalog [10], an online database that compiles data from genomic association studies and offers a curated collection of published GWAS that evaluate at least 100,000 single nucleotide polymorphisms (SNP), revealed a grouping of 15 SNPs associated with obesity in intron 1 of *FTO* gene [11], and a total of 61 different intronic SNPs associated with BMI, body fat distribution and other obesity characteristics, were identified from GWAS, almost all present in Europeans, African/African-Americans, Asians, South Asians and Latino/Admixed Americans (miscegenated populations in Latin America) and, at smaller rates (19/61) in Native Americans (Peruvian Amerindians) [12]. Although the available data indicate that the SNPS associated with obesity are located in the first intron of the *FTO* gene, it is important to understand exon mutations to evaluate their effects not only on obesity, but also on other genetic diseases, as indicated by some studies. For example, a rare, non-synonymous exonic mutation (p.Arg322Gln) has been associated with congenital malformations in two siblings from a Yemen inbreeding family [13] whereas another rare non-synonymous exonic mutation (p.Arg316Gln) has been associated with a lethal autosomal recessive syndrome, resulting in normal development impairment of the central nervous and cardiovascular systems [14]. The *FTO* p.Ala134Thr variant has been associated with leukopenia induced by thiopurine, related to Inflammatory Bowel Disease [15] and two missense variants (p.Cys326Ser and p.Ser256Asn) were associated to reduced semen quality [16]. Another missense mutation in the *FTO* gene has been associated with microcephaly, developmental delay, behavioral abnormalities, dysmorphic facial features, hypotonia and several other phenotypic abnormalities in a five-year-old girl born from an inbreeding marriage [17]. At present, complete sequencing data for the exome of continental populations are available at public databases, such as the Genome Aggregation Database (gnomAD), 1000 Genomes and the NHLBI Exome Sequencing Project (Exome Variant Server), providing an excellent opportunity to investigate the pathogenicity of these mutations and their potentially functional alleles in the *FTO* gene. In this context, the aim of this study was to track nonsynonymous variants in *FTO* gene exons in different population groups using the gnomAD database and analyze the potential functional effects of these variants.

## Methodology

*FTO* gene data available at the gnomAD 2.1 were downloaded on May 1, 2021 from https://gnomad.broadinstitute.org/. The GnomAD, also known as the Genome Aggregation Database Consortium, was developed by an international coalition of researchers to aggregate and harmonize exome and genome sequencing data from a wide range of large-scale sequencing projects and make data summaries available for the scientific community. Formerly known as the Exome Aggregation Consortium (ExAC), the project began in 2012 and expanded on the work of the 1000 Genomes Project and others that cataloged human genetic variations [18, 19]. The reference genome used for sequence alignment was GRCh37/hg19 (reference), and alignment was performed using the GATK tool [20]. Variants were analyzed using the following publicly available pathogenicity prediction programs: FATHMM [21], PROVEAN [22], SIFT [23], POLYPHEN-2 [24] and PANTHER [25]. The variants subjected to predictor analysis followed this conformation, exclusively: p.Ala405Val, p.Tyr23Cys, p.Ser256Asn and so on. Synonymous mutations were excluded from the analyses, as well as variants in intronic regions. The c.-60C>T, c.-56dupG, c.-48C>T mutations were also excluded. The criteria employed to classify the nature of the mutations were as follows: benign, when three or more predictors classified the variant as benign; pathogenic, when three or more predictors classified the mutation as pathogenic; inconclusive, when at least one predictor was unable to analyze the variant, two classified it as pathogenic and two others classified it as benign or when no prediction was made by multiple predictors.

The ClinVar [26] is one of the most commonly applied databases for clinical and pathological mutation analysis. Although the vast majority of mutations are not reported in this database, those considered pathogenic or benign will be subjected to a search to support the findings of this study or published literature reports.

## Results

In total, 345 nonsynonymous mutations were identified at the gnomAD database, of which nineteen were stop-gain mutations (5.6%), 321 were missense mutations (93%) and five were splice region mutations (1.4%). Of the 345 identified mutations, 134 (38.8%) were classified as pathogenic, 144 (41.7%) were classified as benign, and 67 (19.5%) were classified as inconclusive based on *in silico* analyses by five pathogenicity predictors (Table 1).

**Table 1:** Number of types of mutations and their characterizations according to the employed predictors.

Type of mutation		Pathogenicity (Five predictors) *	
Missense	321 (93%)	Damage	134 (38.8%)
Stop Gained	19 (5.6%)	Benign	144 (41,7%)
Splice Region	5 (1.4%)	Unknown	67 (19.5%)
**Total**	**345 (100%)**	**Total**	**345 (100%)**

* FATHMM, PANTHER, SIFT, PROVEAN and POPLYPHEN-2.

Information on the position, nucleotide change, amino acid change, type of mutation, allele count, number of alleles and frequency of each variant in Latino/Admixed American, South Asian, East Asian, African/African-American, European (non-Finnish) and European (Finnish) populations is presented in S1 Table. Of the 38 mutations identified in South Asians, 27 were classified as pathogenic, 27 as benign and 16 as inconclusive. The most frequent mutation (Arg123Trp), classified as inconclusive, was the only one detected at a frequency ≥ 1% (1.7%). The other mutations were detected at very low frequencies. The most common pathogenic mutation was Glu325Val (0.69%). In East Asian populations, 45 very rare mutations were identified, 12 classified as pathogenic, 22 as benign and eleven as inconclusive. The most common mutation, p.Ala134Thr (0.02), was classified as benign. The variants classified as pathogenic exhibited frequencies in the range of 0.0001. Eighty-eight mutations with very low frequencies, most in the range of 1/10,000 or more, were detected in African/African-American populations. Of these, 25 were classified as pathogenic, 42 as benign and 21 as inconclusive. In this population, three mutations exhibited frequencies greater than 1%, namely p.Ala405Val, p.Tyr23Cys and p.Gly182Ala estimated, respectively, at 0.02, 0.04 and 0.01. In Europeans (non-Finnish), a greater number of mutations was detected (188), but all displaying with very low frequencies, most in the range of 1/10,000 or more. Of these, 69 were classified as pathogenic, 82 as benign and 37 as inconclusive. In Europeans (Finnish), on the other hand, only 21 variants were found, 12 classified as benign, six as pathogenic and three classified as inconclusive. The most common variant, p.Asp332Gly, classified as pathogenic, exhibited a frequency of 0.005, while the other variants displayed frequencies in the range of 0.0001. A total of 75 rare mutations were identified in Latino/Admixed American populations, 30 classified as pathogenic, 29 as benign and 14 as inconclusive. The most common variant in Latino/Admixed American populations was p.Ser256Asn (0.002), classified as benign. Globally, the most common variant, p.Tyr23Cys, was found in 773 individuals (0.003), most common in African/African-Americans (0.021) and also detected in Europeans (Non-Finnish) and Latino/Admixed Americans. However, the pathogenicity of this variant was classified as inconclusive by all five predictors employed herein (S2 Table). A total of 12 variants were detected in the “Ashkenazi” Jew population, a name used to refer to Jews from Central and Eastern Europe, eight of which were benign mutations, one pathogenic and three inconclusive. A benign p.Ala163Thr mutation was found in 70 Ashkenazi Jewish, with a frequency of 0.006.

Among the variants classified as pathogenic, 69 were found in Europeans (non-Finnish), 27 iIn South Asians, 30 in Latino/Admixed American, 25 in African/African-Americans, 12 in East Asians, six in Europeans (Finnish) and only one in Ashkenazi Jewish. Globally, the most common pathogenic variant was the p.Glu325Val substitution (0.0008), found with a frequency of 0.006 in South Asians and detected at a very low frequency in Latino/Admixed Americans,. Sixty-five pathogenic mutations are shared by more than one population group, 17 of which are found in Europeans and in one or two other continental populations, suggesting a European origin for this variant, spread by migration, as follows: variants p.Arg84Ser, p.Pro117Ser, p.Tyr333Cys, p.Pro399Ala and p.His62Arg for Latino/Admixed American, p.Pro93Leu, p.Arg445Cys and p.Arg322Ter for Latino/Admixed Americans and South Asians, p.Val65Phe, p.Arg322Gln and p.Arg388Ter for South Asians, p.Cys338Arg and p.Thr115Met for East Asians, African/African-Americans and Ashkenazi Jewish, p.Gln306Lys, p.Met207Val and p.Pro93Arg for African/African-Americans, and p.Asn143Ser for Latins, African/African-Americans and South Asians.

## Discussion

**Fig 1:**
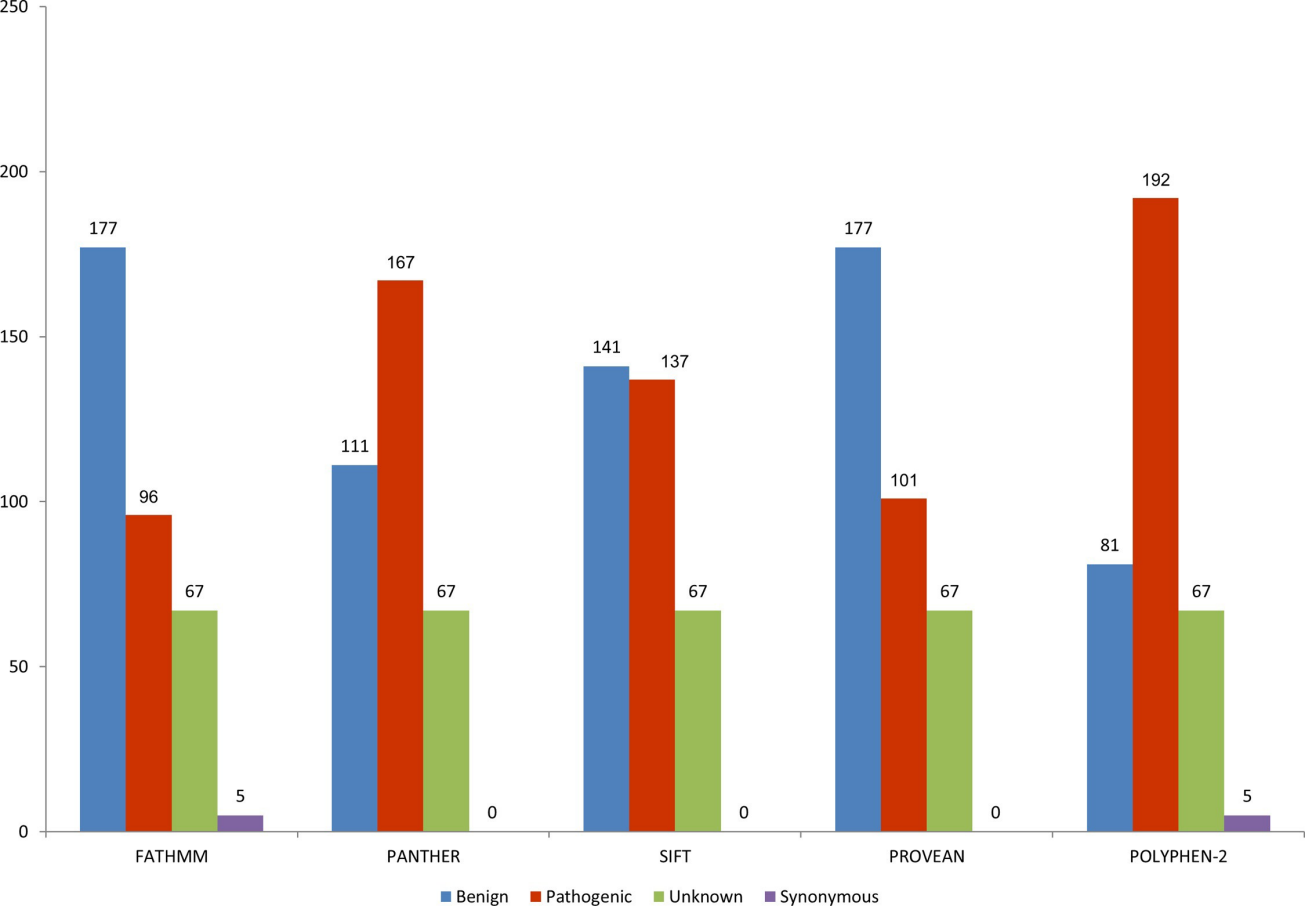
Performance (%) of the employed pathogenicity prediction programs in the analysis of 158 nonsynonymous variants found at the gnomAD database.

As expected, divergences between the pathogenicity analysis findings concerning the variants performed by the five prediction programs employed herein were observed (S3 Table). In general, both the FATHMM and PROVEAN programs classified most variants as benign (177; 51.3%), while the SIFT, POLYPHEN and PANTHER programs classified most variants as pathogenic (137; 39.7%, 192; 55.6% and 167; 48.4%, respectively (Fig 1).

Pathogenicity prediction programs allow for the evaluation of the effect of amino acid substitutions on protein structure or function without performing functional studies, and the available data indicate that the average accuracy of pathogenicity predictors is 85%. However, as different pathogenicity prediction programs vary widely in their methods and ability to predict the pathogenicity of a given sequence change, significant disagreements in the identification of mutational effects and pathogenicity among different programs are noted [27, 28]. In total, 67 exonic variants were classified as pathogenic by all five predictors employed in this study (S3 Table). Exonic *FTO* gene variants have been little explored, and the few studies available to screen for variants by exon sequencing of *FTO* gene have found no evidence that the identified variants confer an increased risk of obesity. A total of 34 variants were identified on obese European children (English, French, Belgian and Swiss), [29], but only seven non-synonymous variants were found in Chinese (Han) children with early-onset obesity [30] and four in obese African/African-American children [31]. Likewise, next-generation sequencing (NGS) of the *FTO* gene in severely obese Swedish children has revealed little evidence of functional variants in the coding region of this gene [32]. These data corroborate the suggestion that the *FTO* gene does not exert its effects on BMI and obesity through functional mutations, and that this effect is more likely to be exerted by the intron 1 of the *FTO* gene regulating the expression of the *IRX3* and *IRX5* loci, vital for adipocyte maturation [6, 7].

The findings of complete exome sequencing data from large populations available at the Genome Aggregation Database (gnomAD) indicate a substantial number of rare coding variants classified as pathogenic or potentially pathogenic by different pathogenicity prediction programs which are not detected by GWAS due to low linkage disequilibrium, as well as the GWAS limitations in capturing rare variants present in less than 1.0% of the investigated population. However, the available data [29–31] suggest that these variants are probably not associated with BMI and obesity but instead, with other diseases [13–17]. Functional studies are, this, required to identify the role of these variants in disease genesis.

The obvious limitation of this work is that it does not explore the non-synonymous exonic variants identified at the gene expression level in an attempt to identify the biological effects underlying these 134 potentially pathogenic mutations, which constitutes a challenge to be addressed in due course. Additionally, these variants should be explored in an exome database of indigenous and non-indigenous Brazilians, which are not included in the gnomAD.

## Supporting information

S1 Table Missense variants in the FTO gene found in the gnomAD database by population. FATHMM, PANTHER, SIFT, PROVEAN and POLYPHEN-2.
(DOCX)

S2 Table Missense variants in the FTO gene found in the gnomAD database in the global population. * Five predictors: FATHMM, PANTHER, SIFT, PROVEAN and POLYPHEN-2.
(DOCX)

S3 Table Missense variants in the FTO gene found in the gnomAD database and pathogenicity based on five predictor programs. (DOCX)
