# Supplementary material for: Analysis of coding variants in the human FTO gene from the gnomAD database
Source: PLoS One. 2022 Jan 6;17(1):e0248610. doi: 10.1371/journal.pone.0248610 (PMC8735611; doi:10.1371/journal.pone.0248610)
Supplement: S1 Table — FATHMM, PANTHER, SIFT, PROVEAN and POLYPHEN-2. (DOCX) [file pone.0248610.s001.docx]

S1 Table. Missense variants in the FTO gene found in the gnomAD database by population.

| **Population** | **Position** | **rsID** | **Substitution** | **Pathogenicity^a^** | **Allele Count** | **Allele Number** | **Allele Frequency** |
| --- | --- | --- | --- | --- | --- | --- | --- |
| African/African-American | 53922838 | rs16952624 | p.Ala405Val | Benign | 738 | 24970 | 0,029555467 |
| African/African-American | 53945359 | rs80130403 | p.Tyr23Cys | Unknown | 716 | 15178 | 0,047173541 |
| African/African-American | 53860197 | rs61743972 | p.Gly182Ala | Benign | 336 | 24970 | 0,013456147 |
| African/African-American | 54145767 | rs150820365 | p.Arg64His | Unknown | 20 | 24966 | 0,000801089 |
| African/African-American | 53913861 | rs369714221 | p.Pro361Ser | Benign | 18 | 24944 | 0,000721616 |
| African/African-American | 53945367 | rs761594967 | p.Ile26Val | Unknown | 18 | 15184 | 0,001185458 |
| African/African-American | 53860139 | rs145884431 | p.Ala163Thr | Benign | 12 | 24964 | 0,000480692 |
| African/African-American | 53913823 | rs149659678 | p.Asp348Val | Benign | 12 | 24956 | 0,000480846 |
| African/African-American | 54145859 | rs781021746 | p.Arg95Gly | Unknown | 11 | 24952 | 0,000440846 |
| African/African-American | 53945317 | rs144100465 | p.Cys9Tyr | Unknown | 10 | 15238 | 0,000656254 |
| African/African-American | 53878082 | rs144743617 | p.Ser256Asn | Benign | 9 | 24970 | 0,000360433 |
| African/African-American | 54145774 | rs370137051 | p.Leu489Phe | Pathogenic | 9 | 24966 | 0,00036049 |
| African/African-American | 53860080 | rs147561986 | p.Asn143Ser | Pathogenic | 8 | 24966 | 0,000320436 |
| African/African-American | 53860029 | rs374337023 | p.Lys126Arg | Benign | 8 | 24962 | 0,000320487 |
| African/African-American | 53967931 | rs376527078 | p.Gly425Val | Benign | 7 | 24964 | 0,000280404 |
| African/African-American | 54145784 | rs138241079 | p.Ile492Thr | Benign | 7 | 24964 | 0,000280404 |
| African/African-American | 53945376 | rs941300321 | p.Gly29Arg | Unknown | 5 | 15184 | 0,000329294 |
| African/African-American | 53878102 | rs200452822 | p.Glu263Lys | Benign | 4 | 24956 | 0,000160282 |
| African/African-American | 53945364 | rs932706316 | p.Cys25Arg | Unknown | 4 | 15194 | 0,000263262 |
| African/African-American | 53945349 | rs762828848 | p.Lys20Glu | Unknown | 4 | 15194 | 0,000263262 |
| African/African-American | 54145822 | rs566271902 | p.Pro505Thr | Benign | 4 | 24958 | 0,000160269 |
| African/African-American | 53860052 | rs79206939 | p.Ala134Thr | Benign | 3 | 24966 | 0,000120163 |
| African/African-American | 54145786 | rs763703220 | p.Val493Ile | Benign | 3 | 24968 | 0,000120154 |
| African/African-American | 53860271 | rs138348216 | p.Met207Val | Pathogenic | 3 | 24974 | 0,000120125 |
| African/African-American | 54145848 | rs377384000 | p.Arg91Gln | Unknown | 3 | 16256 | 0,000184547 |
| African/African-American | 53878130 | rs756523431 | p.Val272Ala | Pathogenic | 3 | 24960 | 0,000120192 |
| African/African-American | 54145741 | rs529673094 | p.Lys478Glu | Benign | 3 | 16256 | 0,000184547 |
| African/African-American | 54145868 | rs1489246528 | p.Leu98Ile | Pathogenic | 3 | 24924 | 0,000120366 |
| African/African-American | 53860253 | rs150450891 | p.Val201Ile | Benign | 2 | 24968 | 8,01025E-05 |
| African/African-American | 53860088 | rs182784714 | p.Leu146Met | Pathogenic | 2 | 24966 | 8,01089E-05 |
| African/African-American | 53859930 | rs151263395 | p.Pro93Arg | Pathogenic | 2 | 24966 | 8,01089E-05 |
| African/African-American | 53860373 | rs138816516 | p.Ala241Thr | Benign | 2 | 24970 | 8,00961E-05 |
| African/African-American | 54145785 | rs376687583 | p.Ser70Leu | Unknown | 2 | 24968 | 8,01025E-05 |
| African/African-American | 54145694 | rs779678337 | p.Arg462Gln | Benign | 2 | 24968 | 8,01025E-05 |
| African/African-American | 53860331 | rs774078678 | p.Ala227Ser | Benign | 2 | 24970 | 8,00961E-05 |
| African/African-American | 53945308 | rs866111415 | p.Val6Ala | Unknown | 2 | 15232 | 0,000131303 |
| African/African-American | 53844052 | rs774232694 | p.Lys16Gln | Pathogenic | 2 | 16216 | 0,000123335 |
| African/African-American | 53844100 | rs373028121 | p.Thr32Ser | Benign | 2 | 24948 | 8,01667E-05 |
| African/African-American | 53878094 | rs76762929 | p.Ser260Tyr | Benign | 2 | 16256 | 0,000123031 |
| African/African-American | 53913810 | rs141978030 | p.Asn344Asp | Pathogenic | 2 | 24958 | 8,01346E-05 |
| African/African-American | 54145834 | rs778420628 | p.Ser86Arg | Unknown | 2 | 16256 | 0,000123031 |
| African/African-American | 54145962 | rs1182749892 | p.Ser129Asn | Unknown | 2 | 8708 | 0,000229674 |
| African/African-American | 54145943 | rs533303860 | p.Arg123Trp | Unknown | 1 | 22444 | 4,45553E-05 |
| African/African-American | 54145952 | rs567718105 | p.Ile126Val | Unknown | 1 | 21982 | 4,54918E-05 |
| African/African-American | 54145727 | rs777486753 | p.Arg473Gln | Benign | 1 | 24960 | 4,00641E-05 |
| African/African-American | 53859890 | rs140101381 | p.Arg80Trp | Pathogenic | 1 | 24958 | 4,00673E-05 |
| African/African-American | 54145783 | rs373102373 | p.Ile492Val | Benign | 1 | 24968 | 4,00513E-05 |
| African/African-American | 53922746 | rs145529071 | p.Val374Val | Benign | 1 | 24968 | 4,00513E-05 |
| African/African-American | 53967939 | rs371704660 | p.Val428Met | Benign | 1 | 24968 | 4,00513E-05 |
| African/African-American | 53967952 | rs141327394 | p.Asn432Ser | Benign | 1 | 16256 | 6,15157E-05 |
| African/African-American | 54145847 | rs758033988 | p.Arg91Trp | Unknown | 1 | 16256 | 6,15157E-05 |
| African/African-American | 53913789 | rs368490949 | p.Arg337Cys | Pathogenic | 1 | 24964 | 4,00577E-05 |
| African/African-American | 53860010 | rs774450343 | p.Val120Met | Benign | 1 | 16256 | 6,15157E-05 |
| African/African-American | 53738118 | rs778741837 | p.Glu8Lys | Benign | 1 | 17424 | 5,73921E-05 |
| African/African-American | 53967979 | rs558995583 | p.Ser441Leu | Benign | 1 | 16256 | 6,15157E-05 |
| African/African-American | 54145693 | rs141983035 | p.Arg462Ter | Benign | 1 | 16256 | 6,15157E-05 |
| African/African-American | 54145747 | rs1172821402 | p.Asp480Asn | Benign | 1 | 16256 | 6,15157E-05 |
| African/African-American | 53860047 | rs141423836 | p.Ile132Thr | Benign | 1 | 16254 | 6,15233E-05 |
| African/African-American | 53907718 | rs1350889567 | p.Gln306Lys | Pathogenic | 1 | 24964 | 4,00577E-05 |
| African/African-American | 53967913 | rs371906409 | p.His419Arg | Benign | 1 | 16256 | 6,15157E-05 |
| African/African-American | 54145685 | rs937565568 | p.Arg459Gln | Benign | 1 | 24966 | 4,00545E-05 |
| African/African-American | 53738129 | rs1388384491 | p.Glu11Asp | Pathogenic | 1 | 8716 | 0,000114732 |
| African/African-American | 53738136 | rs1016221892 | p.Ala14Thr | Pathogenic | 1 | 8714 | 0,000114758 |
| African/African-American | 53844082 | rs1294612571 | p.Thr26Ala | Benign | 1 | 8710 | 0,000114811 |
| African/African-American | 53844122 | rs769612021 | p.Tyr39Cys | Pathogenic | 1 | 8718 | 0,000114705 |
| African/African-American | 53859800 | rs148579300 | p.Ile50Leu | Benign | 1 | 15648 | 6,39059E-05 |
| African/African-American | 53859825 |  | p.Ser58Tyr | Pathogenic | 1 | 16232 | 6,16067E-05 |
| African/African-American | 53859941 | rs368658799 | p.Ile97Val | Benign | 1 | 16256 | 6,15157E-05 |
| African/African-American | 53860016 | rs867050060 | p.Gly122Trp | Pathogenic | 1 | 8712 | 0,000114784 |
| African/African-American | 53860096 | rs141915969 | p.Ile148Met | Benign | 1 | 16256 | 6,15157E-05 |
| African/African-American | 53860122 | rs1400616279 | p.Leu157Pro | Pathogenic | 1 | 8708 | 0,000114837 |
| African/African-American | 53860262 | rs760541418 | p.Leu204Met | Pathogenic | 1 | 16254 | 6,15233E-05 |
| African/African-American | 53860298 | rs1028237381 | p.Lys216Gln | Pathogenic | 1 | 16256 | 6,15157E-05 |
| African/African-American | 53860300 | rs370009039 | p.Lys216Asn | Pathogenic | 1 | 16256 | 6,15157E-05 |
| African/African-American | 53878094 | rs76762929 | p.Ser260Cys | Benign | 1 | 16256 | 6,15157E-05 |
| African/African-American | 53878171 | rs376957531 | p.Ala286Thr | Benign | 1 | 16256 | 6,15157E-05 |
| African/African-American | 53907724 | rs542365655 | p.Cys308Arg | Pathogenic | 1 | 16256 | 6,15157E-05 |
| African/African-American | 53913758 | rs1287663418 | p.Cys326Cys | Benign | 1 | 8702 | 0,000114916 |
| African/African-American | 53913769 | rs773329068 | p.Thr330Ile | Pathogenic | 1 | 16256 | 6,15157E-05 |
| African/African-American | 53913829 | rs377073096 | p.Asp350Gly | Benign | 1 | 16256 | 6,15157E-05 |
| African/African-American | 53922805 | rs1441842690 | p.Asp394Gly | Pathogenic | 1 | 16256 | 6,15157E-05 |
| African/African-American | 53945304 | rs925727658 | p.Lys5Gln | Unknown | 1 | 8710 | 0,000114811 |
| African/African-American | 53945326 | rs1366935034 | p.Val12Ala | Unknown | 1 | 6502 | 0,000153799 |
| African/African-American | 53945328 | rs1008775419 | p.Glu13Lys | Pathogenic | 1 | 6502 | 0,000153799 |
| African/African-American | 53945361 | rs1424620579 | p.Arg24Cys | Unknown | 1 | 8714 | 0,000114758 |
| African/African-American | 53945374 | rs777553242 | p.His28Pro | Unknown | 1 | 6470 | 0,00015456 |
| African/African-American | 53967918 | rs748820946 | p.Val421Ile | Benign | 1 | 16254 | 6,15233E-05 |
| African/African-American | 54145949 | rs748318601 | p.Pro125Ser | Unknown | 1 | 13476 | 7,4206E-05 |
| Latino/Admixed American | 53878082 | rs144743617 | p.Ser256Asn | Benign | 79 | 35414 | 0,002230756 |
| Latino/Admixed American | 53860139 | rs145884431 | p.Ala163Thr | Benign | 70 | 35440 | 0,001975169 |
| Latino/Admixed American | 53859837 | rs550932456 | p.His62Arg | Pathogenic | 46 | 35436 | 0,001298115 |
| Latino/Admixed American | 53945359 | rs80130403 | p.Tyr23Cys | Unknown | 45 | 25322 | 0,001777111 |
| Latino/Admixed American | 53922838 | rs16952624 | p.Ala405Val | Benign | 36 | 35436 | 0,001015916 |
| Latino/Admixed American | 53860197 | rs61743972 | p.Gly182Ala | Benign | 32 | 35438 | 0,000902985 |
| Latino/Admixed American | 53859904 | rs753462430 | p.Arg84Ser | Pathogenic | 26 | 34592 | 0,000751619 |
| Latino/Admixed American | 53945317 | rs144100465 | p.Cys9Tyr | Unknown | 25 | 25322 | 0,000987284 |
| Latino/Admixed American | 53860088 | rs182784714 | p.Leu146Met | Pathogenic | 22 | 35428 | 0,000620978 |
| Latino/Admixed American | 53859890 | rs140101381 | p.Arg80Trp | Pathogenic | 13 | 35440 | 0,000366817 |
| Latino/Admixed American | 54145881 | rs552046216 | p.Asn102Ser | Unknown | 12 | 34564 | 0,000347182 |
| Latino/Admixed American | 54145952 | rs567718105 | p.Ile126Val | Unknown | 7 | 31380 | 0,000223072 |
| Latino/Admixed American | 53878172 | rs370075174 | p.Ala286Val | Benign | 7 | 35420 | 0,000197628 |
| Latino/Admixed American | 53945331 | rs1360239441 | p.Pro14Ser | Unknown | 6 | 24474 | 0,000245158 |
| Latino/Admixed American | 54145702 | rs754711298 | p.Pro465Ser | Pathogenic | 6 | 34592 | 0,000173451 |
| Latino/Admixed American | 54145872 | rs1217207612 | p.Phe99Ser | Unknown | 6 | 34576 | 0,000173531 |
| Latino/Admixed American | 54145767 | rs150820365 | p.Arg64His | Unknown | 5 | 35440 | 0,000141084 |
| Latino/Admixed American | 54145783 | rs373102373 | p.Ile492Val | Benign | 5 | 35440 | 0,000141084 |
| Latino/Admixed American | 53922746 | rs145529071 | p.Val374Val | Benign | 5 | 35436 | 0,000141099 |
| Latino/Admixed American | 53738106 | rs752817421 | p.Thr4Pro | Benign | 4 | 24750 | 0,000161616 |
| Latino/Admixed American | 53878127 | rs868433402 | p.His271Arg | Benign | 4 | 34572 | 0,000115701 |
| Latino/Admixed American | 54145727 | rs777486753 | p.Arg473Gln | Benign | 3 | 35438 | 8,46549E-05 |
| Latino/Admixed American | 53967952 | rs141327394 | p.Asn432Ser | Benign | 3 | 34586 | 8,67403E-05 |
| Latino/Admixed American | 54145866 | rs375406387 | p.Arg97Gln | Unknown | 3 | 35428 | 8,46788E-05 |
| Latino/Admixed American | 53859930 | rs151263395 | p.Pro93Leu | Pathogenic | 3 | 35438 | 8,46549E-05 |
| Latino/Admixed American | 53913797 | rs200895945 | p.Gln339His | Benign | 3 | 35440 | 8,46501E-05 |
| Latino/Admixed American | 53922819 | rs776456069 | p.Pro399Ser | Pathogenic | 3 | 34588 | 8,67353E-05 |
| Latino/Admixed American | 53913861 | rs369714221 | p.Pro361Ser | Benign | 2 | 35426 | 5,64557E-05 |
| Latino/Admixed American | 53878102 | rs200452822 | p.Glu263Lys | Benign | 2 | 35418 | 5,64685E-05 |
| Latino/Admixed American | 53945364 | rs932706316 | p.Cys25Arg | Unknown | 2 | 25322 | 7,89827E-05 |
| Latino/Admixed American | 53860253 | rs150450891 | p.Val201Ile | Benign | 2 | 35438 | 5,64366E-05 |
| Latino/Admixed American | 54145943 | rs533303860 | p.Arg123Trp | Unknown | 2 | 33202 | 6,02373E-05 |
| Latino/Admixed American | 53907776 | rs202007463 | p.Glu325Val | Pathogenic | 2 | 34524 | 5,79307E-05 |
| Latino/Admixed American | 53738107 | rs1456216323 | p.Thr4Ile | Benign | 2 | 24750 | 8,08081E-05 |
| Latino/Admixed American | 53844101 | rs1194621251 | p.Thr32Ile | Pathogenic | 2 | 34574 | 5,78469E-05 |
| Latino/Admixed American | 53859894 | rs764576608 | p.Asp81Gly | Pathogenic | 2 | 34592 | 5,78168E-05 |
| Latino/Admixed American | 53859968 | rs1479729827 | p.Tyr106His | Pathogenic | 2 | 34586 | 5,78269E-05 |
| Latino/Admixed American | 53860178 | rs1473976471 | p.Phe176Ile | Benign | 2 | 34592 | 5,78168E-05 |
| Latino/Admixed American | 53860202 | rs772503437 | p.Ser184Pro | Benign | 2 | 34590 | 5,78202E-05 |
| Latino/Admixed American | 53878175 | rs1290922677 | p.Ile287Thr | Benign | 2 | 34570 | 5,78536E-05 |
| Latino/Admixed American | 53968021 | rs755686716 | p.Arg455Lys | Pathogenic | 2 | 34576 | 5,78436E-05 |
| Latino/Admixed American | 53945367 | rs761594967 | p.Ile26Val | Unknown | 1 | 25322 | 3,94914E-05 |
| Latino/Admixed American | 53860080 | rs147561986 | p.Asn143Ser | Pathogenic | 1 | 35430 | 2,82247E-05 |
| Latino/Admixed American | 53860029 | rs374337023 | p.Lys126Arg | Benign | 1 | 35438 | 2,82183E-05 |
| Latino/Admixed American | 53860052 | rs79206939 | p.Ala134Thr | Benign | 1 | 35430 | 2,82247E-05 |
| Latino/Admixed American | 53860331 | rs774078678 | p.Ala227Ser | Benign | 1 | 35438 | 2,82183E-05 |
| Latino/Admixed American | 53913789 | rs368490949 | p.Arg337Cys | Pathogenic | 1 | 35438 | 2,82183E-05 |
| Latino/Admixed American | 54145685 | rs937565568 | p.Arg459Gln | Benign | 1 | 35440 | 2,82167E-05 |
| Latino/Admixed American | 53913778 | rs781481485 | p.Tyr333Cys | Pathogenic | 1 | 34592 | 2,89084E-05 |
| Latino/Admixed American | 53967990 | rs758990618 | p.Arg445Cys | Pathogenic | 1 | 34584 | 2,89151E-05 |
| Latino/Admixed American | 53907766 | rs200201735 | p.Arg322Ter | Pathogenic | 1 | 35396 | 2,82518E-05 |
| Latino/Admixed American | 53738103 | rs765157936 | p.Arg3Cys | Pathogenic | 1 | 24750 | 4,0404E-05 |
| Latino/Admixed American | 53844083 | rs756522457 | p.Thr26Ile | Benign | 1 | 34574 | 2,89235E-05 |
| Latino/Admixed American | 53859807 | rs755828209 | p.Arg52Gln | Benign | 1 | 34582 | 2,89168E-05 |
| Latino/Admixed American | 53907748 | rs777196899 | p.Arg316Trp | Pathogenic | 1 | 34568 | 2,89285E-05 |
| Latino/Admixed American | 53922819 | rs776456069 | p.Pro399Ala | Pathogenic | 1 | 34588 | 2,89118E-05 |
| Latino/Admixed American | 53945362 | rs755788528 | p.Arg24His | Unknown | 1 | 24474 | 4,08597E-05 |
| Latino/Admixed American | 54145754 | rs1401317361 | p.Arg60Ter | Unknown | 1 | 34592 | 2,89084E-05 |
| Latino/Admixed American | 53738102 | rs989161287 | p.Lys2Asn | Pathogenic | 1 | 24750 | 4,0404E-05 |
| Latino/Admixed American | 53844120 | rs780803760 | p.Phe38Leu | Pathogenic | 1 | 34566 | 2,89302E-05 |
| Latino/Admixed American | 53859864 | rs772076301 | p.Thr71Ile | Pathogenic | 1 | 34592 | 2,89084E-05 |
| Latino/Admixed American | 53859899 | rs773635184 | p.Val83Phe | Pathogenic | 1 | 34592 | 2,89084E-05 |
| Latino/Admixed American | 53859984 | rs1191932724 | p.Thr111Asn | Pathogenic | 1 | 34590 | 2,89101E-05 |
| Latino/Admixed American | 53860001 | rs759168981 | p.Pro117Ser | Pathogenic | 1 | 34592 | 2,89084E-05 |
| Latino/Admixed American | 53860362 | rs1192563467 | p.Val237Ala | Pathogenic | 1 | 34592 | 2,89084E-05 |
| Latino/Admixed American | 53878115 | rs1475617180 | p.Pro267His | Benign | 1 | 34574 | 2,89235E-05 |
| Latino/Admixed American | 53907751 | rs1370044478 | p.Phe317Ile | Pathogenic | 1 | 34558 | 2,89369E-05 |
| Latino/Admixed American | 53907766 | rs200201735 | p.Arg322Gly | Pathogenic | 1 | 34548 | 2,89452E-05 |
| Latino/Admixed American | 53913756 | rs779204175 | p.Cys326Arg | Pathogenic | 1 | 34592 | 2,89084E-05 |
| Latino/Admixed American | 53945320 | rs75097003 | p.Asn10Ser | Unknown | 1 | 24476 | 4,08563E-05 |
| Latino/Admixed American | 53967950 | rs1488347423 | p.Arg431Ser | Benign | 1 | 34584 | 2,89151E-05 |
| Latino/Admixed American | 53968019 | rs747478505 | p.Ala454Ala | Benign | 1 | 34572 | 2,89251E-05 |
| Latino/Admixed American | 54145813 | rs1476316498 | p.Glu502Gln | Benign | 1 | 34592 | 2,89084E-05 |
| Latino/Admixed American | 54145824 | rs1424370756 | p.Pro83Arg | Unknown | 1 | 34590 | 2,89101E-05 |
| Latino/Admixed American | 54145887 | rs1388976674 | p.Val104Ala | Unknown | 1 | 34554 | 2,89402E-05 |
| Ashkenazi Jewish | 53860139 | rs145884431 | p.Ala163Thr | Benign | 70 | 10366 | 0,006752846 |
| Ashkenazi Jewish | 53945317 | rs144100465 | p.Cys9Tyr | Unknown | 59 | 8608 | 0,006854089 |
| Ashkenazi Jewish | 53878172 | rs370075174 | p.Ala286Val | Benign | 18 | 10366 | 0,001736446 |
| Ashkenazi Jewish | 54145767 | rs150820365 | p.Arg64His | Unknown | 13 | 10370 | 0,001253616 |
| Ashkenazi Jewish | 53878082 | rs144743617 | p.Ser256Asn | Benign | 10 | 10366 | 0,000964692 |
| Ashkenazi Jewish | 53860253 | rs150450891 | p.Val201Ile | Benign | 6 | 10370 | 0,000578592 |
| Ashkenazi Jewish | 54145727 | rs777486753 | p.Arg473Gln | Benign | 1 | 10370 | 9,6432E-05 |
| Ashkenazi Jewish | 54145943 | rs533303860 | p.Arg123Trp | Unknown | 1 | 9598 | 0,000104188 |
| Ashkenazi Jewish | 53860052 | rs79206939 | p.Ala134Thr | Benign | 1 | 10364 | 9,64878E-05 |
| Ashkenazi Jewish | 53913789 | rs368490949 | p.Arg337Cys | Pathogenic | 1 | 10366 | 9,64692E-05 |
| Ashkenazi Jewish | 53738106 | rs752817421 | p.Thr4Ala | Benign | 1 | 8508 | 0,000117536 |
| Ashkenazi Jewish | 53860142 | rs1231516982 | p.Asn164His | Benign | 1 | 10076 | 9,92457E-05 |
| East Asian | 53860052 | rs79206939 | p.Ala134Thr | Benign | 551 | 19950 | 0,027619048 |
| East Asian | 53860082 | rs201086068 | p.Asp144Asn | Benign | 55 | 19944 | 0,002757722 |
| East Asian | 54145915 | rs565387719 | p.Asp113Glu | Unknown | 28 | 19628 | 0,001426534 |
| East Asian | 53859947 | rs775904851 | p.Ile99Val | Benign | 5 | 18394 | 0,000271828 |
| East Asian | 53859879 | rs776743009 | p.Gly76Asp | Pathogenic | 4 | 18394 | 0,000217462 |
| East Asian | 53913789 | rs368490949 | p.Arg337Cys | Pathogenic | 3 | 19950 | 0,000150376 |
| East Asian | 54145943 | rs533303860 | p.Arg123Trp | Unknown | 2 | 18718 | 0,000106849 |
| East Asian | 54145847 | rs758033988 | p.Arg91Trp | Unknown | 2 | 18392 | 0,000108743 |
| East Asian | 54145883 | rs571869687 | p.Val103Ile | Unknown | 2 | 18364 | 0,000108909 |
| East Asian | 53860211 | rs577569584 | p.Gly187Arg | Benign | 2 | 19952 | 0,000100241 |
| East Asian | 53738125 | rs575883122 | p.Arg10Leu | Benign | 2 | 11306 | 0,000176897 |
| East Asian | 53859938 | rs757311078 | p.Arg96Cys | Pathogenic | 2 | 19952 | 0,000100241 |
| East Asian | 53738100 | rs201836578 | p.Lys2Glu | Pathogenic | 2 | 11306 | 0,000176897 |
| East Asian | 53860161 | rs754065579 | p.Leu170Trp | Benign | 2 | 18392 | 0,000108743 |
| East Asian | 53860206 | rs1312261216 | p.Tyr185Cys | Benign | 2 | 18394 | 0,000108731 |
| East Asian | 53738106 | rs752817421 | p.Thr4Ala | Benign | 1 | 11302 | 8,84799E-05 |
| East Asian | 54145881 | rs552046216 | p.Asn102Ser | Unknown | 1 | 18368 | 5,44425E-05 |
| East Asian | 54145786 | rs763703220 | p.Val493Ile | Benign | 1 | 19954 | 5,01153E-05 |
| East Asian | 54145848 | rs377384000 | p.Arg91Gln | Unknown | 1 | 18392 | 5,43715E-05 |
| East Asian | 54145694 | rs779678337 | p.Arg462Gln | Benign | 1 | 19954 | 5,01153E-05 |
| East Asian | 53967979 | rs558995583 | p.Ser441Leu | Benign | 1 | 18368 | 5,44425E-05 |
| East Asian | 53967913 | rs371906409 | p.His419Arg | Benign | 1 | 18364 | 5,44544E-05 |
| East Asian | 53860037 | rs751544648 | p.Glu129Lys | Benign | 1 | 18390 | 5,43774E-05 |
| East Asian | 53859803 | rs371489995 | p.Leu51Ile | Benign | 1 | 19920 | 5,02008E-05 |
| East Asian | 54145865 | rs775623288 | p.Arg97Trp | Unknown | 1 | 18392 | 5,43715E-05 |
| East Asian | 53859996 | rs762343983 | p.Thr115Met | Pathogenic | 1 | 18390 | 5,43774E-05 |
| East Asian | 53913790 | rs761330347 | p.Arg337His | Pathogenic | 1 | 18392 | 5,43715E-05 |
| East Asian | 53913792 | rs766971002 | p.Cys338Arg | Pathogenic | 1 | 18392 | 5,43715E-05 |
| East Asian | 53844103 | rs1292607982 | p.Pro33Thr | Pathogenic | 1 | 18390 | 5,43774E-05 |
| East Asian | 53859870 | rs778065726 | p.His73Arg | Benign | 1 | 18394 | 5,43656E-05 |
| East Asian | 53859881 | rs770061754 | p.Cys77Arg | Pathogenic | 1 | 18394 | 5,43656E-05 |
| East Asian | 53860100 | rs775311728 | p.Thr150Ala | Benign | 1 | 18392 | 5,43715E-05 |
| East Asian | 53860104 | rs1322199273 | p.Ile151Thr | Benign | 1 | 18394 | 5,43656E-05 |
| East Asian | 53860233 | rs1257170214 | p.Lys194Arg | Benign | 1 | 18394 | 5,43656E-05 |
| East Asian | 53878096 | rs1486647785 | p.His261Tyr | Benign | 1 | 18384 | 5,43951E-05 |
| East Asian | 53913802 | rs1284248706 | p.Ala341Asp | Pathogenic | 1 | 18392 | 5,43715E-05 |
| East Asian | 53913880 | rs1435299421 | p.Gly367Glu | Benign | 1 | 18334 | 5,45435E-05 |
| East Asian | 53922812 | rs201572715 | p.Trp396Ter | Pathogenic | 1 | 18384 | 5,43951E-05 |
| East Asian | 53945313 | rs1189932504 | p.Glu8Lys | Benign | 1 | 10526 | 9,50029E-05 |
| East Asian | 53945362 | rs755788528 | p.Arg24Leu | Unknown | 1 | 10518 | 9,50751E-05 |
| East Asian | 53945374 | rs777553242 | p.His28Leu | Unknown | 1 | 10516 | 9,50932E-05 |
| East Asian | 53968008 | rs1399267613 | p.Glu451Gln | Pathogenic | 1 | 18372 | 5,44307E-05 |
| East Asian | 54145771 | rs1487911815 | p.Asp488Asn | Benign | 1 | 1560 | 0,000641026 |
| East Asian | 54145937 | rs745847276 | p.Ser121Gly | Unknown | 1 | 17558 | 5,69541E-05 |
| East Asian | 54145944 | rs1159857501 | p.Arg123Gln | Unknown | 1 | 17126 | 5,83908E-05 |
| Finnish | 53913775 | rs201510895 | p.Asp332Gly | Pathogenic | 127 | 25120 | 0,005055732 |
| Finnish | 53860080 | rs147561986 | p.Asn143Ser | Pathogenic | 37 | 25124 | 0,001472695 |
| Finnish | 53878082 | rs144743617 | p.Ser256Asn | Benign | 33 | 25116 | 0,001313903 |
| Finnish | 53913756 | rs779204175 | p.Cys326Ser | Benign | 11 | 25120 | 0,000437898 |
| Finnish | 53860139 | rs145884431 | p.Ala163Thr | Benign | 7 | 25122 | 0,00027864 |
| Finnish | 54145952 | rs567718105 | p.Ile126Val | Unknown | 7 | 19122 | 0,00036607 |
| Finnish | 53860373 | rs138816516 | p.Ala241Thr | Benign | 6 | 25114 | 0,000238911 |
| Finnish | 53860220 | rs762749778 | p.Glu190Lys | Benign | 3 | 25124 | 0,000119408 |
| Finnish | 54145883 | rs571869687 | p.Val103Ile | Unknown | 2 | 21394 | 9,34842E-05 |
| Finnish | 53945317 | rs144100465 | p.Cys9Tyr | Unknown | 2 | 10430 | 0,000191755 |
| Finnish | 53967939 | rs371704660 | p.Val428Met | Benign | 2 | 25116 | 7,96305E-05 |
| Finnish | 54145703 | rs761068832 | p.Pro465His | Pathogenic | 2 | 25124 | 7,96052E-05 |
| Finnish | 53913789 | rs368490949 | p.Arg337Cys | Pathogenic | 1 | 25120 | 3,98089E-05 |
| Finnish | 53859938 | rs757311078 | p.Arg96Cys | Pathogenic | 1 | 25120 | 3,98089E-05 |
| Finnish | 53860253 | rs150450891 | p.Val201Ile | Benign | 1 | 25122 | 3,98057E-05 |
| Finnish | 54145693 | rs141983035 | p.Arg462Ter | Benign | 1 | 21648 | 4,61936E-05 |
| Finnish | 53922830 | rs765180226 | p.Gln402His | Benign | 1 | 25124 | 3,98026E-05 |
| Finnish | 53859960 | rs1203776934 | p.Gly103Asp | Pathogenic | 1 | 21648 | 4,61936E-05 |
| Finnish | 53860241 | rs774050560 | p.Ala197Thr | Benign | 1 | 21648 | 4,61936E-05 |
| Finnish | 53878186 | rs1223419681 | p.Gln291Ter | Benign | 1 | 3464 | 0,000288684 |
| Finnish | 53967916 | rs1222652247 | p.Glu420Gly | Benign | 1 | 3472 | 0,000288018 |
| European | 53878082 | rs144743617 | p.Ser256Asn | Benign | 487 | 129076 | 0,003772971 |
| European | 53860139 | rs145884431 | p.Ala163Thr | Benign | 338 | 129166 | 0,002616788 |
| European | 53945317 | rs144100465 | p.Cys9Tyr | Unknown | 282 | 69448 | 0,004060592 |
| European | 53860253 | rs150450891 | p.Val201Ile | Benign | 134 | 129144 | 0,001037601 |
| European | 53860080 | rs147561986 | p.Asn143Ser | Pathogenic | 75 | 129132 | 0,000580801 |
| European | 54145952 | rs567718105 | p.Ile126Val | Unknown | 68 | 111902 | 0,000607675 |
| European | 53860088 | rs182784714 | p.Leu146Met | Pathogenic | 56 | 129136 | 0,000433651 |
| European | 53859930 | rs151263395 | p.Pro93Arg | Pathogenic | 31 | 129180 | 0,000239975 |
| European | 54145943 | rs533303860 | p.Arg123Trp | Unknown | 19 | 115830 | 0,000164033 |
| European | 53878172 | rs370075174 | p.Ala286Val | Benign | 19 | 129090 | 0,000147184 |
| European | 54145866 | rs375406387 | p.Arg97Gln | Unknown | 19 | 128512 | 0,000147846 |
| European | 53913775 | rs201510895 | p.Asp332Gly | Pathogenic | 18 | 129094 | 0,000139433 |
| European | 53859890 | rs140101381 | p.Arg80Trp | Pathogenic | 14 | 129182 | 0,000108374 |
| European | 53860373 | rs138816516 | p.Ala241Thr | Benign | 13 | 127066 | 0,000102309 |
| European | 54145727 | rs777486753 | p.Arg473Gln | Benign | 13 | 129186 | 0,00010063 |
| European | 54145783 | rs373102373 | p.Ile492Val | Benign | 13 | 129186 | 0,00010063 |
| European | 53859939 | rs139577103 | p.Arg96His | Pathogenic | 12 | 113750 | 0,000105495 |
| European | 53967939 | rs371704660 | p.Val428Met | Benign | 11 | 129104 | 8,52026E-05 |
| European | 53922746 | rs145529071 | p.Val374Val | Benign | 11 | 129146 | 8,51749E-05 |
| European | 53922838 | rs16952624 | p.Ala405Val | Benign | 10 | 129160 | 7,74234E-05 |
| European | 54145767 | rs150820365 | p.Arg64His | Unknown | 9 | 129188 | 6,96659E-05 |
| European | 53913778 | rs781481485 | p.Tyr333Cys | Pathogenic | 9 | 113678 | 7,9171E-05 |
| European | 54145859 | rs781021746 | p.Arg95Gly | Unknown | 9 | 128754 | 6,99007E-05 |
| European | 53878067 | rs141920596 | p.Gly251Asp | Benign | 9 | 129050 | 6,97404E-05 |
| European | 53913756 | rs779204175 | p.Cys326Ser | Benign | 8 | 129066 | 6,19838E-05 |
| European | 53860052 | rs79206939 | p.Ala134Thr | Benign | 8 | 129118 | 6,19588E-05 |
| European | 53860197 | rs61743972 | p.Gly182Ala | Benign | 8 | 129174 | 6,1932E-05 |
| European | 54145786 | rs763703220 | p.Val493Phe | Benign | 8 | 129176 | 6,1931E-05 |
| European | 53859899 | rs773635184 | p.Val83Leu | Benign | 7 | 113758 | 6,15341E-05 |
| European | 53878088 | rs369122168 | p.Asp258Gly | Benign | 7 | 113672 | 6,15807E-05 |
| European | 53945374 | rs777553242 | p.His28Arg | Unknown | 7 | 69302 | 0,000101007 |
| European | 54145883 | rs571869687 | p.Val103Ile | Unknown | 6 | 112270 | 5,34426E-05 |
| European | 53878102 | rs200452822 | p.Glu263Lys | Benign | 6 | 129070 | 4,64864E-05 |
| European | 53860010 | rs774450343 | p.Val120Met | Benign | 6 | 113724 | 5,27593E-05 |
| European | 53860319 | rs780601119 | p.Met223Val | Pathogenic | 6 | 112392 | 5,33846E-05 |
| European | 53878100 | rs148528422 | p.Leu262Pro | Benign | 6 | 113678 | 5,27807E-05 |
| European | 54145905 | rs1397588632 | p.Ser110Thr | Unknown | 6 | 125854 | 4,76743E-05 |
| European | 53945359 | rs80130403 | p.Tyr23Cys | Unknown | 5 | 69302 | 7,2148E-05 |
| European | 53878158 | rs778545208 | p.Glu281Asp | Benign | 5 | 113680 | 4,39831E-05 |
| European | 53859803 | rs371489995 | p.Leu51Ile | Benign | 4 | 128736 | 3,10713E-05 |
| European | 53967952 | rs141327394 | p.Asn432Ser | Benign | 4 | 113708 | 3,51778E-05 |
| European | 53859939 | rs139577103 | p.Arg96Pro | Pathogenic | 4 | 113750 | 3,51648E-05 |
| European | 53878108 | rs756019558 | p.Arg265Gly | Pathogenic | 4 | 129104 | 3,09828E-05 |
| European | 53913787 | rs760025896 | p.Gln336Pro | Benign | 4 | 113688 | 3,5184E-05 |
| European | 53913853 | rs921941344 | p.Ser358Phe | Pathogenic | 4 | 113698 | 3,51809E-05 |
| European | 53860211 | rs577569584 | p.Gly187Arg | Benign | 3 | 129170 | 2,32252E-05 |
| European | 53859837 | rs550932456 | p.His62Arg | Pathogenic | 3 | 129166 | 2,32259E-05 |
| European | 53859930 | rs151263395 | p.Pro93Leu | Pathogenic | 3 | 129180 | 2,32234E-05 |
| European | 53859915 | rs1410999299 | p.Lys88Arg | Benign | 3 | 113752 | 2,63732E-05 |
| European | 53907767 | rs745616565 | p.Arg322Gln | Pathogenic | 3 | 113550 | 2,64201E-05 |
| European | 53860040 | rs745951679 | p.Ala130Pro | Benign | 3 | 113694 | 2,63866E-05 |
| European | 53907777 | rs749212983 | p.Glu325Glu | Benign | 3 | 113484 | 2,64354E-05 |
| European | 53922747 | rs752687224 | p.Glu375Lys | Pathogenic | 3 | 113726 | 2,63792E-05 |
| European | 53922802 | rs769206684 | p.Thr393Ile | Pathogenic | 3 | 113698 | 2,63857E-05 |
| European | 53967918 | rs748820946 | p.Val421Phe | Benign | 3 | 113628 | 2,64019E-05 |
| European | 54145721 | rs771859785 | p.Glu471Gly | Benign | 3 | 129178 | 2,32238E-05 |
| European | 54145794 | rs767891166 | p.Asn73Ser | Unknown | 3 | 129180 | 2,32234E-05 |
| European | 54145941 | rs756070602 | p.Thr122Ile | Unknown | 3 | 101248 | 2,96302E-05 |
| European | 54145847 | rs758033988 | p.Arg91Trp | Unknown | 2 | 113562 | 1,76115E-05 |
| European | 53913797 | rs200895945 | p.Gln339His | Benign | 2 | 129120 | 1,54895E-05 |
| European | 53738118 | rs778741837 | p.Glu8Lys | Benign | 2 | 73884 | 2,70695E-05 |
| European | 53859781 | rs778691805 | p.Gln43His | Benign | 2 | 128142 | 1,56077E-05 |
| European | 53738112 | rs913398301 | p.Thr6Ala | Benign | 2 | 58662 | 3,40936E-05 |
| European | 53860282 | rs752812594 | p.Gln210His | Benign | 2 | 113548 | 1,76137E-05 |
| European | 53860321 | rs748772299 | p.Met223Ile | Pathogenic | 2 | 112362 | 1,77996E-05 |
| European | 53878068 | rs770905300 | p.Gly251Gly | Benign | 2 | 113628 | 1,76013E-05 |
| European | 53878072 | rs759400907 | p.Glu253Lys | Benign | 2 | 113638 | 1,75997E-05 |
| European | 53878106 | rs757723391 | p.Gly264Asp | Benign | 2 | 113678 | 1,75936E-05 |
| European | 53878118 | rs1164310772 | p.Asp268Gly | Benign | 2 | 129104 | 1,54914E-05 |
| European | 53878193 | rs1239366948 | p.Asp293Val | Pathogenic | 2 | 113662 | 1,7596E-05 |
| European | 53913801 | rs755738675 | p.Ala341Ser | Pathogenic | 2 | 113688 | 1,7592E-05 |
| European | 53913801 | rs755738675 | p.Ala341Thr | Pathogenic | 2 | 113688 | 1,7592E-05 |
| European | 53913874 | rs758583500 | p.Lys365Arg | Benign | 2 | 113680 | 1,75932E-05 |
| European | 53967973 | rs755099738 | p.Leu439Arg | Pathogenic | 2 | 113702 | 1,75898E-05 |
| European | 53967991 | rs139000284 | p.Arg445His | Pathogenic | 2 | 129124 | 1,5489E-05 |
| European | 53968002 | rs1362586808 | p.Arg449Trp | Pathogenic | 2 | 129122 | 1,54892E-05 |
| European | 54145674 | rs376381270 | p.Arg455Ser | Pathogenic | 2 | 113332 | 1,76473E-05 |
| European | 54145851 | rs770709958 | p.Arg92Lys | Unknown | 2 | 113538 | 1,76152E-05 |
| European | 54145883 | rs571869687 | p.Val103Phe | Unknown | 2 | 112270 | 1,78142E-05 |
| European | 54145928 | rs1487229294 | p.Leu118Ile | Unknown | 2 | 121366 | 1,64791E-05 |
| European | 54145941 | rs756070602 | p.Thr122Ser | Unknown | 2 | 101248 | 1,97535E-05 |
| European | 53913789 | rs368490949 | p.Arg337Cys | Pathogenic | 1 | 129118 | 7,74485E-06 |
| European | 54145693 | rs141983035 | p.Arg462Ter | Benign | 1 | 113630 | 8,80049E-06 |
| European | 53922830 | rs765180226 | p.Gln402His | Benign | 1 | 129170 | 7,74174E-06 |
| European | 53859947 | rs775904851 | p.Ile99Val | Benign | 1 | 113754 | 8,7909E-06 |
| European | 53738106 | rs752817421 | p.Thr4Ala | Benign | 1 | 58714 | 1,70317E-05 |
| European | 54145786 | rs763703220 | p.Val493Ile | Benign | 1 | 129176 | 7,74138E-06 |
| European | 54145848 | rs377384000 | p.Arg91Gln | Unknown | 1 | 113566 | 8,80545E-06 |
| European | 54145694 | rs779678337 | p.Arg462Gln | Benign | 1 | 129094 | 7,74629E-06 |
| European | 53859996 | rs762343983 | p.Thr115Met | Pathogenic | 1 | 113718 | 8,79368E-06 |
| European | 53913792 | rs766971002 | p.Cys338Arg | Pathogenic | 1 | 113692 | 8,79569E-06 |
| European | 53859904 | rs753462430 | p.Arg84Ser | Pathogenic | 1 | 113762 | 8,79028E-06 |
| European | 53967990 | rs758990618 | p.Arg445Cys | Pathogenic | 1 | 113704 | 8,79477E-06 |
| European | 53907766 | rs200201735 | p.Arg322Ter | Pathogenic | 1 | 128988 | 7,75266E-06 |
| European | 53922819 | rs776456069 | p.Pro399Ala | Pathogenic | 1 | 113720 | 8,79353E-06 |
| European | 53945362 | rs755788528 | p.Arg24His | Unknown | 1 | 53874 | 1,85618E-05 |
| European | 54145754 | rs1401317361 | p.Arg60Ter | Unknown | 1 | 113764 | 8,79013E-06 |
| European | 53860271 | rs138348216 | p.Met207Val | Pathogenic | 1 | 129084 | 7,74689E-06 |
| European | 54145747 | rs1172821402 | p.Asp480Asn | Benign | 1 | 113766 | 8,78997E-06 |
| European | 53860047 | rs141423836 | p.Ile132Thr | Benign | 1 | 113702 | 8,79492E-06 |
| European | 53907718 | rs1350889567 | p.Gln306Lys | Pathogenic | 1 | 129122 | 7,74461E-06 |
| European | 54145684 | rs755836957 | p.Arg459Ter | Benign | 1 | 113506 | 8,81011E-06 |
| European | 53859845 | rs1304763607 | p.Val65Phe | Pathogenic | 1 | 129168 | 7,74186E-06 |
| European | 53922786 | rs746048206 | p.Arg388Ter | Pathogenic | 1 | 113692 | 8,79569E-06 |
| European | 53945323 | rs769538802 | p.Ser11Thr | Unknown | 1 | 53990 | 1,85219E-05 |
| European | 53945336 | rs1418448100 | p.Cys15Ter | Unknown | 1 | 69360 | 1,44175E-05 |
| European | 54145703 | rs761068832 | p.Pro465Leu | Pathogenic | 1 | 129120 | 7,74473E-06 |
| European | 54145755 | rs770852343 | p.Arg60Gln | Unknown | 1 | 113764 | 8,79013E-06 |
| European | 53738110 | rs1035019229 | p.Pro5Leu | Benign | 1 | 58682 | 1,7041E-05 |
| European | 53738119 | rs1329981588 | p.Glu8Gly | Benign | 1 | 58488 | 1,70975E-05 |
| European | 53738124 | rs1282501161 | p.Arg10Ter | Benign | 1 | 58388 | 1,71268E-05 |
| European | 53844054 |  | p.Lys16Asn | Pathogenic | 1 | 113594 | 8,80328E-06 |
| European | 53844094 | rs1275818602 | p.Tyr30His | Pathogenic | 1 | 113650 | 8,79894E-06 |
| European | 53844114 | rs7499606 | p.Asp36Glu | Benign | 1 | 113640 | 8,79972E-06 |
| European | 53844115 | rs756946176 | p.Glu37Ter | Benign | 1 | 113622 | 8,80111E-06 |
| European | 53859778 | rs768675500 | p.Trp42Ter | Pathogenic | 1 | 112624 | 8,8791E-06 |
| European | 53859794 | rs765590518 | p.Lys48Gln | Benign | 1 | 113100 | 8,84173E-06 |
| European | 53859803 | rs371489995 | p.Leu51Phe | Benign | 1 | 113310 | 8,82535E-06 |
| European | 53859820 | rs779539101 | p.Ser56Arg | Benign | 1 | 113666 | 8,79771E-06 |
| European | 53859855 | rs555319581 | p.Ala68Gly | Pathogenic | 1 | 113748 | 8,79136E-06 |
| European | 53859857 | rs1352866890 | p.Phe69Leu | Pathogenic | 1 | 113754 | 8,7909E-06 |
| European | 53859860 | rs748099982 | p.Leu70Phe | Pathogenic | 1 | 113750 | 8,79121E-06 |
| European | 53859877 | rs1256195889 | p.His75Gln | Pathogenic | 1 | 113760 | 8,79044E-06 |
| European | 53859905 | rs1434557313 | p.Ile85Phe | Benign | 1 | 113762 | 8,79028E-06 |
| European | 53859909 | rs778585710 | p.Gln86Pro | Benign | 1 | 113760 | 8,79044E-06 |
| European | 53859977 | rs769204154 | p.Leu109Val | Pathogenic | 1 | 113746 | 8,79152E-06 |
| European | 53859987 | rs1392454854 | p.Arg112Thr | Pathogenic | 1 | 113732 | 8,7926E-06 |
| European | 53860039 | rs757157849 | p.Glu129Asp | Benign | 1 | 113704 | 8,79477E-06 |
| European | 53860105 | rs762625200 | p.Ile151Met | Benign | 1 | 113718 | 8,79368E-06 |
| European | 53860118 | rs751353294 | p.Glu156Lys | Benign | 1 | 113726 | 8,79306E-06 |
| European | 53860144 | rs1332447878 | p.Asn164Lys | Benign | 1 | 15428 | 6,48172E-05 |
| European | 53860157 | rs756242925 | p.Pro169Thr | Pathogenic | 1 | 113748 | 8,79136E-06 |
| European | 53860157 | rs756242925 | p.Pro169Ser | Benign | 1 | 113748 | 8,79136E-06 |
| European | 53860179 | rs779309219 | p.Phe176Cys | Benign | 1 | 113750 | 8,79121E-06 |
| European | 53860184 | rs1407999347 | p.Arg178Gly | Benign | 1 | 113752 | 8,79105E-06 |
| European | 53860222 | rs1282396852 | p.Glu190Asp | Benign | 1 | 113754 | 8,7909E-06 |
| European | 53860229 | rs1212033225 | p.Ile193Val | Benign | 1 | 113754 | 8,7909E-06 |
| European | 53860244 | rs761651552 | p.Ala198Thr | Benign | 1 | 113740 | 8,79198E-06 |
| European | 53860270 | rs753902912 | p.Phe206Leu | Benign | 1 | 113656 | 8,79848E-06 |
| European | 53860271 | rs138348216 | p.Met207Leu | Pathogenic | 1 | 113652 | 8,79879E-06 |
| European | 53860281 | rs779165603 | p.Gln210Arg | Benign | 1 | 113568 | 8,8053E-06 |
| European | 53860334 | rs1179039850 | p.Val228Leu | Pathogenic | 1 | 112114 | 8,91949E-06 |
| European | 53860344 | rs771907956 | p.His231Arg | Pathogenic | 1 | 111900 | 8,93655E-06 |
| European | 53860371 | rs760665708 | p.Ser240Ter | Pathogenic | 1 | 111638 | 8,95752E-06 |
| European | 53878070 | rs776580855 | p.Pro252Arg | Pathogenic | 1 | 113636 | 8,80003E-06 |
| European | 53878114 | rs1016337772 | p.Pro267Thr | Benign | 1 | 113682 | 8,79647E-06 |
| European | 53878157 | rs1380871124 | p.Glu281Val | Benign | 1 | 113696 | 8,79538E-06 |
| European | 53878177 | rs777382917 | p.Pro288Thr | Pathogenic | 1 | 113688 | 8,796E-06 |
| European | 53878199 | rs1259762053 | p.Tyr295Cys | Pathogenic | 1 | 113674 | 8,79709E-06 |
| European | 53878210 | rs777558537 | p.Asp299His | Pathogenic | 1 | 113574 | 8,80483E-06 |
| European | 53907712 | rs766767212 | p.Thr304Ala | Pathogenic | 1 | 113702 | 8,79492E-06 |
| European | 53907740 | rs1462889039 | p.Ser313Leu | Benign | 1 | 113670 | 8,7974E-06 |
| European | 53907742 | rs139814987 | p.Gln314Lys | Benign | 1 | 113660 | 8,79817E-06 |
| European | 53907749 | rs121918214 | p.Arg316Gln | Pathogenic | 1 | 113622 | 8,80111E-06 |
| European | 53907758 | rs781028867 | p.Ser319Tyr | Pathogenic | 1 | 113602 | 8,80266E-06 |
| European | 53913832 | rs1178634936 | p.Asn351Ser | Benign | 1 | 113714 | 8,79399E-06 |
| European | 53922771 | rs747080793 | p.Trp383Arg | Pathogenic | 1 | 113714 | 8,79399E-06 |
| European | 53922778 | rs757468268 | p.Gln385Pro | Pathogenic | 1 | 113702 | 8,79492E-06 |
| European | 53922783 | rs781495764 | p.Asn387Asp | Benign | 1 | 113714 | 8,79399E-06 |
| European | 53922798 | rs1465157191 | p.Cys392Arg | Pathogenic | 1 | 113694 | 8,79554E-06 |
| European | 53945301 | rs1205507227 | p.Arg4Gly | Unknown | 1 | 54074 | 1,84932E-05 |
| European | 53945328 | rs1008775419 | p.Glu13Ter | Pathogenic | 1 | 15422 | 6,48424E-05 |
| European | 53945370 | rs1280940657 | p.His27Tyr | Unknown | 1 | 53886 | 1,85577E-05 |
| European | 53945381 | rs992422634 | p.Lys30Asn | Unknown | 1 | 53856 | 1,8568E-05 |
| European | 53945396 | rs1380126914 | p.Met35Ile | Unknown | 1 | 53832 | 1,85763E-05 |
| European | 53967969 | rs1192322094 | p.Ile438Val | Benign | 1 | 113704 | 8,79477E-06 |
| European | 53967981 |  | p.Leu442Phe | Pathogenic | 1 | 113710 | 8,7943E-06 |
| European | 53968021 | rs755686716 | p.Arg455Met | Pathogenic | 1 | 113652 | 8,79879E-06 |
| European | 54018858 | rs1450294366 | p.Arg30Met | Unknown | 1 | 52802 | 1,89387E-05 |
| European | 54018866 | rs1284787398 | p.Gly33Arg | Unknown | 1 | 52778 | 1,89473E-05 |
| European | 54145677 | rs1333596595 | p.Ala34Val | Unknown | 1 | 113380 | 8,8199E-06 |
| European | 54145708 | rs747801335 | p.Asp467Asn | Pathogenic | 1 | 113728 | 8,79291E-06 |
| European | 54145708 | rs747801335 | p.Asp467Tyr | Pathogenic | 1 | 113728 | 8,79291E-06 |
| European | 54145724 | rs1291247246 | p.Cys472Tyr | Benign | 1 | 15430 | 6,48088E-05 |
| European | 54145726 | rs1254596324 | p.Arg473Trp | Benign | 1 | 113750 | 8,79121E-06 |
| European | 54145743 | rs1412828427 | p.Lys478Asn | Benign | 1 | 113766 | 8,78997E-06 |
| European | 54145766 | rs776819552 | p.Pro486Leu | Pathogenic | 1 | 15430 | 6,48088E-05 |
| European | 54145774 | rs370137051 | p.Leu489Val | Pathogenic | 1 | 113760 | 8,79044E-06 |
| European | 54145796 | rs750897231 | p.Leu496Pro | Pathogenic | 1 | 113764 | 8,79013E-06 |
| European | 54145812 | rs1421215513 | p.Trp79Ter | Unknown | 1 | 15422 | 6,48424E-05 |
| European | 54145813 | rs1476316498 | p.Glu502Ter | Benign | 1 | 15424 | 6,4834E-05 |
| European | 54145841 | rs1393406068 | p.Leu89Phe | Unknown | 1 | 113668 | 8,79755E-06 |
| European | 54145851 | rs770709958 | p.Arg92Met | Unknown | 1 | 113538 | 8,80762E-06 |
| European | 54145857 | rs1216468868 | p.Lys94Arg | Unknown | 1 | 113408 | 8,81772E-06 |
| European | 54145859 | rs781021746 | p.Arg95Ter | Unknown | 1 | 113332 | 8,82363E-06 |
| European | 54145920 | rs757006993 | p.Ser115Phe | Unknown | 1 | 107760 | 9,27988E-06 |
| European | 54145925 | rs1240856803 | p.Pro117Ser | Pathogenic | 1 | 106350 | 9,40291E-06 |
| European | 54145931 | rs1203482144 | p.Asp119Asn | Unknown | 1 | 105286 | 9,49794E-06 |
| South Asian | 54145943 | rs533303860 | p.Arg123Trp | Unknown | 520 | 29300 | 0,01774744 |
| South Asian | 53907776 | rs202007463 | p.Glu325Val | Pathogenic | 214 | 30606 | 0,006992093 |
| South Asian | 54145866 | rs375406387 | p.Arg97Gln | Unknown | 123 | 30616 | 0,004017507 |
| South Asian | 54145952 | rs567718105 | p.Ile126Val | Unknown | 86 | 28018 | 0,003069455 |
| South Asian | 53860139 | rs145884431 | p.Ala163Thr | Benign | 84 | 30616 | 0,002743663 |
| South Asian | 54145727 | rs777486753 | p.Arg473Gln | Benign | 26 | 30616 | 0,000849229 |
| South Asian | 53878082 | rs144743617 | p.Ser256Asn | Benign | 18 | 30614 | 0,000587966 |
| South Asian | 54145796 | rs750897231 | p.Leu496His | Pathogenic | 18 | 30616 | 0,000587928 |
| South Asian | 53860037 | rs751544648 | p.Glu129Lys | Benign | 13 | 30612 | 0,00042467 |
| South Asian | 53860052 | rs79206939 | p.Ala134Thr | Benign | 8 | 30612 | 0,000261335 |
| South Asian | 53945317 | rs144100465 | p.Cys9Tyr | Unknown | 7 | 22492 | 0,000311222 |
| South Asian | 53878207 | rs746623287 | p.Leu298Phe | Pathogenic | 6 | 30608 | 0,000196027 |
| South Asian | 53878172 | rs370075174 | p.Ala286Val | Benign | 4 | 30612 | 0,000130668 |
| South Asian | 54145847 | rs758033988 | p.Arg91Trp | Unknown | 4 | 30616 | 0,000130651 |
| South Asian | 54145865 | rs775623288 | p.Arg97Trp | Unknown | 4 | 30614 | 0,000130659 |
| South Asian | 54145785 | rs376687583 | p.Ser70Leu | Unknown | 4 | 30616 | 0,000130651 |
| South Asian | 53860311 | rs771548627 | p.Tyr220Cys | Pathogenic | 4 | 30616 | 0,000130651 |
| South Asian | 54145767 | rs150820365 | p.Arg64His | Unknown | 3 | 30616 | 9,7988E-05 |
| South Asian | 53738125 | rs575883122 | p.Arg10Leu | Benign | 3 | 22788 | 0,000131648 |
| South Asian | 53967933 | rs576410322 | p.Leu426Phe | Benign | 3 | 30614 | 9,79944E-05 |
| South Asian | 54145791 | rs762447726 | p.Gln72Arg | Unknown | 3 | 30616 | 9,7988E-05 |
| South Asian | 54145883 | rs571869687 | p.Val103Ile | Unknown | 2 | 30584 | 6,53937E-05 |
| South Asian | 54145684 | rs755836957 | p.Arg459Ter | Benign | 2 | 30616 | 6,53253E-05 |
| South Asian | 53844080 | rs572790599 | p.Asp25Gly | Pathogenic | 2 | 30614 | 6,53296E-05 |
| South Asian | 53844100 | rs373028121 | p.Thr32Ala | Pathogenic | 2 | 30612 | 6,53339E-05 |
| South Asian | 53859899 | rs773635184 | p.Val83Ile | Benign | 2 | 30616 | 6,53253E-05 |
| South Asian | 53913898 | rs1340002370 | p.Glu373Val | Pathogenic | 2 | 30550 | 6,54664E-05 |
| South Asian | 53922787 | rs780342015 | p.Arg388Pro | Pathogenic | 2 | 30614 | 6,53296E-05 |
| South Asian | 54145875 | rs774908366 | p.Ser100Phe | Unknown | 2 | 30606 | 6,53467E-05 |
| South Asian | 54145935 | rs753447446 | p.Cys120Tyr | Unknown | 2 | 29882 | 6,69299E-05 |
| South Asian | 53860080 | rs147561986 | p.Asn143Ser | Pathogenic | 1 | 30616 | 3,26627E-05 |
| South Asian | 53967939 | rs371704660 | p.Val428Met | Benign | 1 | 30612 | 3,26669E-05 |
| South Asian | 53922838 | rs16952624 | p.Ala405Val | Benign | 1 | 30616 | 3,26627E-05 |
| South Asian | 53860197 | rs61743972 | p.Gly182Ala | Benign | 1 | 30614 | 3,26648E-05 |
| South Asian | 53878102 | rs200452822 | p.Glu263Lys | Benign | 1 | 30614 | 3,26648E-05 |
| South Asian | 53967952 | rs141327394 | p.Asn432Ser | Benign | 1 | 30612 | 3,26669E-05 |
| South Asian | 53860211 | rs577569584 | p.Gly187Arg | Benign | 1 | 30616 | 3,26627E-05 |
| South Asian | 53859930 | rs151263395 | p.Pro93Leu | Pathogenic | 1 | 30612 | 3,26669E-05 |
| South Asian | 53907767 | rs745616565 | p.Arg322Gln | Pathogenic | 1 | 30612 | 3,26669E-05 |
| South Asian | 54145786 | rs763703220 | p.Val493Ile | Benign | 1 | 30616 | 3,26627E-05 |
| South Asian | 53967990 | rs758990618 | p.Arg445Cys | Pathogenic | 1 | 30614 | 3,26648E-05 |
| South Asian | 53907766 | rs200201735 | p.Arg322Ter | Pathogenic | 1 | 30612 | 3,26669E-05 |
| South Asian | 54145747 | rs1172821402 | p.Asp480Asn | Benign | 1 | 30616 | 3,26627E-05 |
| South Asian | 53859845 | rs1304763607 | p.Val65Phe | Pathogenic | 1 | 30616 | 3,26627E-05 |
| South Asian | 53922786 | rs746048206 | p.Arg388Ter | Pathogenic | 1 | 30614 | 3,26648E-05 |
| South Asian | 53945323 | rs769538802 | p.Ser11Thr | Unknown | 1 | 22492 | 4,44603E-05 |
| South Asian | 53859938 | rs757311078 | p.Arg96Cys | Pathogenic | 1 | 30612 | 3,26669E-05 |
| South Asian | 53860082 | rs201086068 | p.Asp144Asn | Benign | 1 | 30614 | 3,26648E-05 |
| South Asian | 54145881 | rs552046216 | p.Asn102Ser | Unknown | 1 | 30592 | 3,26883E-05 |
| South Asian | 53913790 | rs761330347 | p.Arg337His | Pathogenic | 1 | 30614 | 3,26648E-05 |
| South Asian | 53738103 | rs765157936 | p.Arg3Cys | Pathogenic | 1 | 22786 | 4,38866E-05 |
| South Asian | 53844083 | rs756522457 | p.Thr26Ile | Benign | 1 | 30612 | 3,26669E-05 |
| South Asian | 53859807 | rs755828209 | p.Arg52Gln | Benign | 1 | 30612 | 3,26669E-05 |
| South Asian | 53907748 | rs777196899 | p.Arg316Trp | Pathogenic | 1 | 30612 | 3,26669E-05 |
| South Asian | 53738104 | rs1161946063 | p.Arg3Pro | Pathogenic | 1 | 22786 | 4,38866E-05 |
| South Asian | 53738113 | rs546144833 | p.Thr6Ile | Benign | 1 | 22788 | 4,38827E-05 |
| South Asian | 53859791 | rs759800072 | p.Pro47Ser | Benign | 1 | 30606 | 3,26733E-05 |
| South Asian | 53859845 | rs1304763607 | p.Val65Ile | Benign | 1 | 30616 | 3,26627E-05 |
| South Asian | 53859879 | rs776743009 | p.Gly76Val | Pathogenic | 1 | 30616 | 3,26627E-05 |
| South Asian | 53859897 | rs1417363477 | p.Leu82Pro | Pathogenic | 1 | 30616 | 3,26627E-05 |
| South Asian | 53859976 | rs1253634102 | p.Tyr108Ter | Pathogenic | 1 | 30614 | 3,26648E-05 |
| South Asian | 53860137 | rs1292567537 | p.Lys162Arg | Benign | 1 | 30614 | 3,26648E-05 |
| South Asian | 53860374 | rs753958759 | p.Ala241Val | Benign | 1 | 30614 | 3,26648E-05 |
| South Asian | 53860390 | rs1302917355 | p.Ser246Arg | Pathogenic | 1 | 30614 | 3,26648E-05 |
| South Asian | 53907736 |  | p.Gly312Ser | Pathogenic | 1 | 30612 | 3,26669E-05 |
| South Asian | 53922825 | rs1271268742 | p.Ala401Thr | Benign | 1 | 30614 | 3,26648E-05 |
| South Asian | 53945385 | rs1421133978 | p.Phe32Val | Unknown | 1 | 22490 | 4,44642E-05 |
| South Asian | 54145694 | rs779678337 | p.Glu40Ter | Unknown | 1 | 30616 | 3,26627E-05 |
| South Asian | 54145701 | rs1277348916 | p.Leu464Phe | Pathogenic | 1 | 30616 | 3,26627E-05 |
| South Asian | 54145759 | rs1329766069 | p.Pro484Ser | Pathogenic | 1 | 30616 | 3,26627E-05 |

^a^ FATHMM, PANTHER, SIFT, PROVEAN and POLYPHEN-2
