# Supplementary material for: Analysis of coding variants in the human FTO gene from the gnomAD database
Source: PLoS One. 2022 Jan 6;17(1):e0248610. doi: 10.1371/journal.pone.0248610 (PMC8735611; doi:10.1371/journal.pone.0248610)
Supplement: S2 Table — * Five predictors: FATHMM, PANTHER, SIFT, PROVEAN and POLYPHEN-2. (DOCX) [file pone.0248610.s002.docx]

S2 Table. Missense variants in the FTO gene found in the gnomAD database in the global population.

| **Position** | **rsIDs** | **HGVS Consequence** | **Pathogenicity^a^** | **VEP Annotation** | **Allele Count** | **Allele Number** | **Allele Frequency** |
| --- | --- | --- | --- | --- | --- | --- | --- |
| 53922838 | rs16952624 | p.Ala405Val | Benign | missense_variant | 791 | 282848 | 0,002796555 |
| 53945359 | rs80130403 | p.Tyr23Cys | Unknown | missense_variant | 773 | 168692 | 0,004582316 |
| 53878082 | rs144743617 | p.Ser256Asn | Benign | missense_variant | 656 | 282720 | 0,002320317 |
| 53860139 | rs145884431 | p.Ala163Thr | Benign | missense_variant | 603 | 282854 | 0,002131842 |
| 53860052 | rs79206939 | p.Ala134Thr | Benign | missense_variant | 577 | 282784 | 0,002040427 |
| 54145943 | rs533303860 | p.Arg123Trp | Unknown | missense_variant | 554 | 256020 | 0,002163893 |
| 53945317 | rs144100465 | p.Cys9Tyr | Unknown | missense_variant | 396 | 168922 | 0,002344277 |
| 53860197 | rs61743972 | p.Gly182Ala | Benign | missense_variant | 381 | 282862 | 0,001346947 |
| 53907776 | rs202007463 | p.Glu325Val | Pathogenic | missense_variant | 218 | 250982 | 0,000868588 |
| 54145952 | rs567718105 | p.Ile126Val | Unknown | missense_variant | 175 | 246102 | 0,000711087 |
| 53913775 | rs201510895 | p.Asp332Gly | Pathogenic | missense_variant | 150 | 282754 | 0,000530496 |
| 54145866 | rs375406387 | p.Arg97Gln | Unknown | missense_variant | 150 | 282028 | 0,000531862 |
| 53860253 | rs150450891 | p.Val201Ile | Benign | missense_variant | 147 | 282838 | 0,000519732 |
| 53860080 | rs147561986 | p.Asn143Ser | Pathogenic | missense_variant | 128 | 282810 | 0,000452601 |
| 53860088 | rs182784714 | p.Leu146Met | Pathogenic | missense_variant | 85 | 282808 | 0,000300557 |
| 53860082 | rs201086068 | p.Asp144Asn | Benign | missense_variant | 56 | 282786 | 0,00019803 |
| 53878172 | rs370075174 | p.Ala286Val | Benign | missense_variant | 53 | 282720 | 0,000187465 |
| 54145767 | rs150820365 | p.Arg64His | Unknown | missense_variant | 51 | 282886 | 0,000180285 |
| 53859837 | rs550932456 | p.His62Arg | Pathogenic | missense_variant | 49 | 282842 | 0,000173242 |
| 54145727 | rs777486753 | p.Arg473Gln | Benign | missense_variant | 46 | 282874 | 0,000162617 |
| 53859930 | rs151263395 | p.Pro93Arg | Pathogenic | missense_variant | 34 | 282862 | 0,0001202 |
| 53859890 | rs140101381 | p.Arg80Trp | Pathogenic | missense_variant | 30 | 282868 | 0,000106057 |
| 54145915 | rs565387719 | p.Asp113Glu | Unknown | missense_variant | 28 | 273986 | 0,000102195 |
| 53859904 | rs753462430 | p.Arg84Ser | Pathogenic | missense_variant | 27 | 251484 | 0,000107363 |
| 53913756 | rs779204175 | p.Cys326Ser | Benign | missense_variant | 25 | 282738 | 8,84211E-05 |
| 53860373 | rs138816516 | p.Ala241Thr | Benign | missense_variant | 21 | 280730 | 7,4805E-05 |
| 53913861 | rs369714221 | p.Pro361Ser | Benign | missense_variant | 20 | 282634 | 7,07629E-05 |
| 54145783 | rs373102373 | p.Ile492Val | Benign | missense_variant | 20 | 282882 | 7,07009E-05 |
| 54145796 | rs750897231 | p.Leu496His | Pathogenic | missense_variant | 20 | 251490 | 7,9526E-05 |
| 54145859 | rs781021746 | p.Arg95Gly | Unknown | missense_variant | 20 | 282354 | 7,08331E-05 |
| 53945367 | rs761594967 | p.Ile26Val | Unknown | missense_variant | 19 | 168706 | 0,000112622 |
| 53922746 | rs145529071 | p.Val374Val | Benign | splice_region_variant | 18 | 282832 | 6,3642E-05 |
| 53967939 | rs371704660 | p.Val428Met | Benign | missense_variant | 15 | 282738 | 5,30526E-05 |
| 53860037 | rs751544648 | p.Glu129Lys | Benign | missense_variant | 14 | 251398 | 5,56886E-05 |
| 54145881 | rs552046216 | p.Asn102Ser | Unknown | missense_variant | 14 | 249772 | 5,60511E-05 |
| 53878102 | rs200452822 | p.Glu263Lys | Benign | missense_variant | 13 | 282698 | 4,59855E-05 |
| 53859939 | rs139577103 | p.Arg96His | Pathogenic | missense_variant | 12 | 251466 | 4,77202E-05 |
| 53913823 | rs149659678 | p.Asp348Val | Benign | missense_variant | 12 | 282754 | 4,24397E-05 |
| 54145883 | rs571869687 | p.Val103Ile | Unknown | missense_variant | 12 | 249362 | 4,81228E-05 |
| 53913778 | rs781481485 | p.Tyr333Cys | Pathogenic | missense_variant | 10 | 251392 | 3,97785E-05 |
| 53860029 | rs374337023 | p.Lys126Arg | Benign | missense_variant | 9 | 282830 | 3,18212E-05 |
| 53878067 | rs141920596 | p.Gly251Asp | Benign | missense_variant | 9 | 282670 | 3,18392E-05 |
| 53967952 | rs141327394 | p.Asn432Ser | Benign | missense_variant | 9 | 251386 | 3,58015E-05 |
| 54145774 | rs370137051 | p.Leu489Phe | Pathogenic | missense_variant | 9 | 282890 | 3,18145E-05 |
| 54145847 | rs758033988 | p.Arg91Trp | Unknown | missense_variant | 9 | 251250 | 3,58209E-05 |
| 53913789 | rs368490949 | p.Arg337Cys | Pathogenic | missense_variant | 8 | 282782 | 2,82903E-05 |
| 54145786 | rs763703220 | p.Val493Phe | Benign | missense_variant | 8 | 282876 | 2,82809E-05 |
| 53859899 | rs773635184 | p.Val83Leu | Benign | missense_variant | 7 | 251480 | 2,78352E-05 |
| 53859930 | rs151263395 | p.Pro93Leu | Pathogenic | missense_variant | 7 | 282862 | 2,4747E-05 |
| 53860010 | rs774450343 | p.Val120Met | Benign | missense_variant | 7 | 251430 | 2,78408E-05 |
| 53878088 | rs369122168 | p.Asp258Gly | Benign | missense_variant | 7 | 251360 | 2,78485E-05 |
| 53945364 | rs932706316 | p.Cys25Arg | Unknown | missense_variant | 7 | 168708 | 4,14918E-05 |
| 53945374 | rs777553242 | p.His28Arg | Unknown | missense_variant | 7 | 168684 | 4,14977E-05 |
| 53967931 | rs376527078 | p.Gly425Val | Benign | missense_variant | 7 | 282734 | 2,47583E-05 |
| 54145784 | rs138241079 | p.Ile492Thr | Benign | missense_variant | 7 | 282876 | 2,47458E-05 |
| 54145785 | rs376687583 | p.Ser70Leu | Unknown | missense_variant | 7 | 282874 | 2,4746E-05 |
| 53859947 | rs775904851 | p.Ile99Val | Benign | missense_variant | 6 | 251474 | 2,38593E-05 |
| 53860211 | rs577569584 | p.Gly187Arg | Benign | missense_variant | 6 | 282858 | 2,12121E-05 |
| 53860319 | rs780601119 | p.Met223Val | Pathogenic | missense_variant | 6 | 250094 | 2,3991E-05 |
| 53878100 | rs148528422 | p.Leu262Pro | Benign | missense_variant | 6 | 251368 | 2,38694E-05 |
| 53878207 | rs746623287 | p.Leu298Phe | Pathogenic | missense_variant | 6 | 251266 | 2,38791E-05 |
| 53945331 | rs1360239441 | p.Pro14Ser | Unknown | missense_variant | 6 | 137404 | 4,36669E-05 |
| 54145702 | rs754711298 | p.Pro465Ser | Pathogenic | missense_variant | 6 | 251424 | 2,38641E-05 |
| 54145786 | rs763703220 | p.Val493Ile | Benign | missense_variant | 6 | 282876 | 2,12107E-05 |
| 54145872 | rs1217207612 | p.Phe99Ser | Unknown | missense_variant | 6 | 250366 | 2,39649E-05 |
| 54145905 | rs1397588632 | p.Ser110Thr | Unknown | missense_variant | 6 | 277358 | 2,16327E-05 |
| 53738106 | rs752817421 | p.Thr4Ala | Benign | missense_variant | 5 | 154494 | 3,23637E-05 |
| 53738125 | rs575883122 | p.Arg10Leu | Benign | missense_variant | 5 | 154228 | 3,24195E-05 |
| 53859803 | rs371489995 | p.Leu51Ile | Benign | missense_variant | 5 | 281722 | 1,7748E-05 |
| 53860271 | rs138348216 | p.Met207Val | Pathogenic | missense_variant | 5 | 282780 | 1,76816E-05 |
| 53878158 | rs778545208 | p.Glu281Asp | Benign | missense_variant | 5 | 251368 | 1,98912E-05 |
| 53913797 | rs200895945 | p.Gln339His | Benign | missense_variant | 5 | 282788 | 1,76811E-05 |
| 53945376 | rs941300321 | p.Gly29Arg | Unknown | missense_variant | 5 | 168684 | 2,96412E-05 |
| 54145848 | rs377384000 | p.Arg91Gln | Unknown | missense_variant | 5 | 251248 | 1,99007E-05 |
| 54145865 | rs775623288 | p.Arg97Trp | Unknown | missense_variant | 5 | 250644 | 1,99486E-05 |
| 53738106 | rs752817421 | p.Thr4Pro | Benign | missense_variant | 4 | 154494 | 2,5891E-05 |
| 53859879 | rs776743009 | p.Gly76Asp | Pathogenic | missense_variant | 4 | 251478 | 1,5906E-05 |
| 53859915 | rs1410999299 | p.Lys88Arg | Benign | missense_variant | 4 | 251470 | 1,59065E-05 |
| 53859938 | rs757311078 | p.Arg96Cys | Pathogenic | missense_variant | 4 | 282860 | 1,41413E-05 |
| 53859939 | rs139577103 | p.Arg96Pro | Pathogenic | missense_variant | 4 | 251466 | 1,59067E-05 |
| 53860311 | rs771548627 | p.Tyr220Cys | Pathogenic | missense_variant | 4 | 250506 | 1,59677E-05 |
| 53878108 | rs756019558 | p.Arg265Gly | Pathogenic | missense_variant | 4 | 282754 | 1,41466E-05 |
| 53878127 | rs868433402 | p.His271Arg | Benign | missense_variant | 4 | 251390 | 1,59115E-05 |
| 53907767 | rs745616565 | p.Arg322Gln | Pathogenic | missense_variant | 4 | 251134 | 1,59278E-05 |
| 53913787 | rs760025896 | p.Gln336Pro | Benign | missense_variant | 4 | 251400 | 1,59109E-05 |
| 53913853 | rs921941344 | p.Ser358Phe | Pathogenic | missense_variant | 4 | 251384 | 1,59119E-05 |
| 53945349 | rs762828848 | p.Lys20Glu | Unknown | missense_variant | 4 | 168726 | 2,37071E-05 |
| 53967990 | rs758990618 | p.Arg445Cys | Pathogenic | missense_variant | 4 | 251382 | 1,5912E-05 |
| 54145694 | rs779678337 | p.Arg462Gln | Benign | missense_variant | 4 | 282788 | 1,41449E-05 |
| 54145822 | rs566271902 | p.Pro505Thr | Benign | missense_variant | 4 | 282852 | 1,41417E-05 |
| 53738100 | rs201836578 | p.Lys2Glu | Pathogenic | missense_variant | 3 | 154900 | 1,93673E-05 |
| 53738118 | rs778741837 | p.Glu8Lys | Benign | missense_variant | 3 | 185678 | 1,6157E-05 |
| 53859781 | rs778691805 | p.Gln43His | Benign | missense_variant | 3 | 279790 | 1,07223E-05 |
| 53860040 | rs745951679 | p.Ala130Pro | Benign | missense_variant | 3 | 251396 | 1,19334E-05 |
| 53860220 | rs762749778 | p.Glu190Lys | Benign | missense_variant | 3 | 282884 | 1,06051E-05 |
| 53860331 | rs774078678 | p.Ala227Ser | Benign | missense_variant | 3 | 281224 | 1,06677E-05 |
| 53878130 | rs756523431 | p.Val272Ala | Pathogenic | missense_variant | 3 | 282762 | 1,06096E-05 |
| 53907766 | rs200201735 | p.Arg322Ter | Pathogenic | stop_gained | 3 | 282526 | 1,06185E-05 |
| 53907777 | rs749212983 | p.Glu325Glu | Benign | splice_region_variant | 3 | 250936 | 1,19552E-05 |
| 53922747 | rs752687224 | p.Glu375Lys | Pathogenic | missense_variant | 3 | 251444 | 1,19311E-05 |
| 53922802 | rs769206684 | p.Thr393Ile | Pathogenic | missense_variant | 3 | 251394 | 1,19335E-05 |
| 53922819 | rs776456069 | p.Pro399Ser | Pathogenic | missense_variant | 3 | 251422 | 1,19321E-05 |
| 53945308 | rs866111415 | p.Val6Ala | Unknown | missense_variant | 3 | 168898 | 1,77622E-05 |
| 53967918 | rs748820946 | p.Val421Phe | Benign | missense_variant | 3 | 251292 | 1,19383E-05 |
| 53967933 | rs576410322 | p.Leu426Phe | Benign | missense_variant | 3 | 251352 | 1,19355E-05 |
| 53967979 | rs558995583 | p.Ser441Leu | Benign | missense_variant | 3 | 251380 | 1,19341E-05 |
| 54145684 | rs755836957 | p.Arg459Ter | Benign | stop_gained | 3 | 251230 | 1,19412E-05 |
| 54145693 | rs141983035 | p.Arg462Ter | Benign | stop_gained | 3 | 251354 | 1,19354E-05 |
| 54145721 | rs771859785 | p.Glu471Gly | Benign | missense_variant | 3 | 282872 | 1,06055E-05 |
| 54145741 | rs529673094 | p.Lys478Glu | Benign | missense_variant | 3 | 251492 | 1,19288E-05 |
| 54145747 | rs1172821402 | p.Asp480Asn | Benign | missense_variant | 3 | 251492 | 1,19288E-05 |
| 54145791 | rs762447726 | p.Gln72Arg | Unknown | missense_variant | 3 | 251484 | 1,19292E-05 |
| 54145794 | rs767891166 | p.Asn73Ser | Unknown | missense_variant | 3 | 282876 | 1,06054E-05 |
| 54145868 | rs1489246528 | p.Leu98Ile | Pathogenic | missense_variant | 3 | 281868 | 1,06433E-05 |
| 54145941 | rs756070602 | p.Thr122Ile | Unknown | missense_variant | 3 | 226172 | 1,32642E-05 |
| 53738103 | rs765157936 | p.Arg3Cys | Pathogenic | missense_variant | 2 | 154620 | 1,29349E-05 |
| 53738107 | rs1456216323 | p.Thr4Ile | Benign | missense_variant | 2 | 154506 | 1,29445E-05 |
| 53738112 | rs913398301 | p.Thr6Ala | Benign | missense_variant | 2 | 154470 | 1,29475E-05 |
| 53844052 | rs774232694 | p.Lys16Gln | Pathogenic | missense_variant | 2 | 251188 | 7,96216E-06 |
| 53844080 | rs572790599 | p.Asp25Gly | Pathogenic | missense_variant | 2 | 251306 | 7,95843E-06 |
| 53844083 | rs756522457 | p.Thr26Ile | Benign | missense_variant | 2 | 251300 | 7,95862E-06 |
| 53844100 | rs373028121 | p.Thr32Ser | Benign | missense_variant | 2 | 282684 | 7,07504E-06 |
| 53844100 | rs373028121 | p.Thr32Ala | Pathogenic | missense_variant | 2 | 251306 | 7,95843E-06 |
| 53844101 | rs1194621251 | p.Thr32Ile | Pathogenic | missense_variant | 2 | 251314 | 7,95817E-06 |
| 53859807 | rs755828209 | p.Arg52Gln | Benign | missense_variant | 2 | 250582 | 7,98142E-06 |
| 53859845 | rs1304763607 | p.Val65Phe | Pathogenic | missense_variant | 2 | 282860 | 7,07064E-06 |
| 53859894 | rs764576608 | p.Asp81Gly | Pathogenic | missense_variant | 2 | 251484 | 7,95279E-06 |
| 53859899 | rs773635184 | p.Val83Ile | Benign | missense_variant | 2 | 251480 | 7,95292E-06 |
| 53859968 | rs1479729827 | p.Tyr106His | Pathogenic | missense_variant | 2 | 251456 | 7,95368E-06 |
| 53859996 | rs762343983 | p.Thr115Met | Pathogenic | missense_variant | 2 | 251430 | 7,9545E-06 |
| 53860047 | rs141423836 | p.Ile132Thr | Benign | missense_variant | 2 | 251410 | 7,95513E-06 |
| 53860161 | rs754065579 | p.Leu170Trp | Benign | missense_variant | 2 | 251476 | 7,95305E-06 |
| 53860178 | rs1473976471 | p.Phe176Ile | Benign | missense_variant | 2 | 251474 | 7,95311E-06 |
| 53860202 | rs772503437 | p.Ser184Pro | Benign | missense_variant | 2 | 251466 | 7,95336E-06 |
| 53860206 | rs1312261216 | p.Tyr185Cys | Benign | missense_variant | 2 | 251464 | 7,95342E-06 |
| 53860282 | rs752812594 | p.Gln210His | Benign | missense_variant | 2 | 251266 | 7,95969E-06 |
| 53860321 | rs748772299 | p.Met223Ile | Pathogenic | missense_variant | 2 | 250056 | 7,99821E-06 |
| 53878068 | rs770905300 | p.Gly251Gly | Benign | splice_region_variant | 2 | 251298 | 7,95868E-06 |
| 53878072 | rs759400907 | p.Glu253Lys | Benign | missense_variant | 2 | 251308 | 7,95836E-06 |
| 53878094 | rs76762929 | p.Ser260Tyr | Benign | missense_variant | 2 | 251338 | 7,95741E-06 |
| 53878106 | rs757723391 | p.Gly264Asp | Benign | missense_variant | 2 | 251366 | 7,95653E-06 |
| 53878118 | rs1164310772 | p.Asp268Gly | Benign | missense_variant | 2 | 282760 | 7,07314E-06 |
| 53878175 | rs1290922677 | p.Ile287Thr | Benign | missense_variant | 2 | 251384 | 7,95596E-06 |
| 53878193 | rs1239366948 | p.Asp293Val | Pathogenic | missense_variant | 2 | 251336 | 7,95748E-06 |
| 53907718 | rs1350889567 | p.Gln306Lys | Pathogenic | missense_variant | 2 | 282756 | 7,07324E-06 |
| 53907748 | rs777196899 | p.Arg316Trp | Pathogenic | missense_variant | 2 | 251288 | 7,959E-06 |
| 53913790 | rs761330347 | p.Arg337His | Pathogenic | missense_variant | 2 | 251392 | 7,9557E-06 |
| 53913792 | rs766971002 | p.Cys338Arg | Pathogenic | missense_variant | 2 | 251402 | 7,95539E-06 |
| 53913801 | rs755738675 | p.Ala341Ser | Pathogenic | missense_variant | 2 | 251394 | 7,95564E-06 |
| 53913801 | rs755738675 | p.Ala341Thr | Pathogenic | missense_variant | 2 | 251394 | 7,95564E-06 |
| 53913810 | rs141978030 | p.Asn344Asp | Pathogenic | missense_variant | 2 | 282782 | 7,07259E-06 |
| 53913874 | rs758583500 | p.Lys365Arg | Benign | missense_variant | 2 | 251334 | 7,95754E-06 |
| 53913898 | rs1340002370 | p.Glu373Val | Pathogenic | missense_variant | 2 | 250406 | 7,98703E-06 |
| 53922786 | rs746048206 | p.Arg388Ter | Pathogenic | stop_gained | 2 | 251370 | 7,9564E-06 |
| 53922787 | rs780342015 | p.Arg388Pro | Pathogenic | missense_variant | 2 | 251384 | 7,95596E-06 |
| 53922819 | rs776456069 | p.Pro399Ala | Pathogenic | missense_variant | 2 | 251422 | 7,95475E-06 |
| 53922830 | rs765180226 | p.Gln402His | Benign | missense_variant | 2 | 282856 | 7,07074E-06 |
| 53945323 | rs769538802 | p.Ser11Thr | Unknown | missense_variant | 2 | 137462 | 1,45495E-05 |
| 53945336 | rs1418448100 | p.Cys15Ter | Unknown | stop_gained | 2 | 168768 | 1,18506E-05 |
| 53945362 | rs755788528 | p.Arg24His | Unknown | missense_variant | 2 | 137298 | 1,45669E-05 |
| 53967913 | rs371906409 | p.His419Arg | Benign | missense_variant | 2 | 251290 | 7,95893E-06 |
| 53967973 | rs755099738 | p.Leu439Arg | Pathogenic | missense_variant | 2 | 251388 | 7,95583E-06 |
| 53967991 | rs139000284 | p.Arg445His | Pathogenic | missense_variant | 2 | 282774 | 7,07279E-06 |
| 53968002 | rs1362586808 | p.Arg449Trp | Pathogenic | missense_variant | 2 | 282776 | 7,07274E-06 |
| 53968021 | rs755686716 | p.Arg455Lys | Pathogenic | missense_variant | 2 | 251304 | 7,95849E-06 |
| 54145674 | rs376381270 | p.Arg455Ser | Pathogenic | missense_variant | 2 | 251056 | 7,96635E-06 |
| 54145685 | rs937565568 | p.Arg459Gln | Benign | missense_variant | 2 | 282670 | 7,07539E-06 |
| 54145703 | rs761068832 | p.Pro465His | Pathogenic | missense_variant | 2 | 282808 | 7,07194E-06 |
| 54145703 | rs761068832 | p.Pro465Leu | Pathogenic | missense_variant | 2 | 282808 | 7,07194E-06 |
| 54145754 | rs1401317361 | p.Arg60Ter | Unknown | stop_gained | 2 | 251490 | 7,9526E-06 |
| 54145755 | rs770852343 | p.Arg60Gln | Unknown | missense_variant | 2 | 251490 | 7,9526E-06 |
| 54145834 | rs778420628 | p.Ser86Arg | Unknown | missense_variant | 2 | 251412 | 7,95507E-06 |
| 54145851 | rs770709958 | p.Arg92Lys | Unknown | missense_variant | 2 | 251218 | 7,96121E-06 |
| 54145875 | rs774908366 | p.Ser100Phe | Unknown | missense_variant | 2 | 250188 | 7,99399E-06 |
| 54145883 | rs571869687 | p.Val103Phe | Unknown | missense_variant | 2 | 249362 | 8,02047E-06 |
| 54145928 | rs1487229294 | p.Leu118Ile | Unknown | missense_variant | 2 | 268162 | 7,45818E-06 |
| 54145935 | rs753447446 | p.Cys120Tyr | Unknown | missense_variant | 2 | 231352 | 8,64484E-06 |
| 54145941 | rs756070602 | p.Thr122Ser | Unknown | missense_variant | 2 | 226172 | 8,84283E-06 |
| 54145962 | rs1182749892 | p.Ser129Asn | Unknown | missense_variant | 2 | 31390 | 6,37146E-05 |
| 53738102 | rs989161287 | p.Lys2Asn | Pathogenic | missense_variant | 1 | 154766 | 6,46137E-06 |
| 53738104 | rs1161946063 | p.Arg3Pro | Pathogenic | missense_variant | 1 | 154588 | 6,46881E-06 |
| 53738110 | rs1035019229 | p.Pro5Leu | Benign | missense_variant | 1 | 154472 | 6,47367E-06 |
| 53738113 | rs546144833 | p.Thr6Ile | Benign | missense_variant | 1 | 154514 | 6,47191E-06 |
| 53738119 | rs1329981588 | p.Glu8Gly | Benign | missense_variant | 1 | 154320 | 6,48004E-06 |
| 53738124 | rs1282501161 | p.Arg10Ter | Benign | stop_gained | 1 | 154230 | 6,48382E-06 |
| 53738129 | rs1388384491 | p.Glu11Asp | Pathogenic | missense_variant | 1 | 31402 | 3,18451E-05 |
| 53738136 | rs1016221892 | p.Ala14Thr | Pathogenic | missense_variant | 1 | 31396 | 3,18512E-05 |
| 53844054 |  | p.Lys16Asn | Pathogenic | missense_variant | 1 | 251224 | 3,98051E-06 |
| 53844082 | rs1294612571 | p.Thr26Ala | Benign | missense_variant | 1 | 31396 | 3,18512E-05 |
| 53844094 | rs1275818602 | p.Tyr30His | Pathogenic | missense_variant | 1 | 251320 | 3,97899E-06 |
| 53844103 | rs1292607982 | p.Pro33Thr | Pathogenic | missense_variant | 1 | 251324 | 3,97893E-06 |
| 53844114 | rs7499606 | p.Asp36Glu | Benign | missense_variant | 1 | 251308 | 3,97918E-06 |
| 53844115 | rs756946176 | p.Glu37Ter | Benign | stop_gained | 1 | 251288 | 3,9795E-06 |
| 53844120 | rs780803760 | p.Phe38Leu | Pathogenic | missense_variant | 1 | 251262 | 3,97991E-06 |
| 53844122 | rs769612021 | p.Tyr39Cys | Pathogenic | missense_variant | 1 | 31404 | 3,18431E-05 |
| 53859778 | rs768675500 | p.Trp42Ter | Pathogenic | stop_gained | 1 | 247968 | 4,03278E-06 |
| 53859791 | rs759800072 | p.Pro47Ser | Benign | missense_variant | 1 | 249188 | 4,01303E-06 |
| 53859794 | rs765590518 | p.Lys48Gln | Benign | missense_variant | 1 | 249600 | 4,00641E-06 |
| 53859800 | rs148579300 | p.Ile50Leu | Benign | missense_variant | 1 | 250172 | 3,99725E-06 |
| 53859803 | rs371489995 | p.Leu51Phe | Benign | missense_variant | 1 | 250326 | 3,99479E-06 |
| 53859820 | rs779539101 | p.Ser56Arg | Benign | missense_variant | 1 | 251292 | 3,97943E-06 |
| 53859825 |  | p.Ser58Tyr | Pathogenic | missense_variant | 1 | 251352 | 3,97848E-06 |
| 53859843 | rs778727414 | p.Glu64Gly | Benign | missense_variant | 1 | 251458 | 3,97681E-06 |
| 53859845 | rs1304763607 | p.Val65Ile | Benign | missense_variant | 1 | 251456 | 3,97684E-06 |
| 53859855 | rs555319581 | p.Ala68Gly | Pathogenic | missense_variant | 1 | 251466 | 3,97668E-06 |
| 53859857 | rs1352866890 | p.Phe69Leu | Pathogenic | missense_variant | 1 | 251472 | 3,97659E-06 |
| 53859860 | rs748099982 | p.Leu70Phe | Pathogenic | missense_variant | 1 | 251474 | 3,97655E-06 |
| 53859864 | rs772076301 | p.Thr71Ile | Pathogenic | missense_variant | 1 | 251478 | 3,97649E-06 |
| 53859870 | rs778065726 | p.His73Arg | Benign | missense_variant | 1 | 251486 | 3,97636E-06 |
| 53859877 | rs1256195889 | p.His75Gln | Pathogenic | missense_variant | 1 | 251484 | 3,9764E-06 |
| 53859879 | rs776743009 | p.Gly76Val | Pathogenic | missense_variant | 1 | 251478 | 3,97649E-06 |
| 53859881 | rs770061754 | p.Cys77Arg | Pathogenic | missense_variant | 1 | 251484 | 3,9764E-06 |
| 53859897 | rs1417363477 | p.Leu82Pro | Pathogenic | missense_variant | 1 | 251484 | 3,9764E-06 |
| 53859899 | rs773635184 | p.Val83Phe | Pathogenic | missense_variant | 1 | 251480 | 3,97646E-06 |
| 53859905 | rs1434557313 | p.Ile85Phe | Benign | missense_variant | 1 | 251486 | 3,97636E-06 |
| 53859908 | rs754590859 | p.Gln86Ter | Benign | stop_gained | 1 | 251482 | 3,97643E-06 |
| 53859909 | rs778585710 | p.Gln86Pro | Benign | missense_variant | 1 | 251478 | 3,97649E-06 |
| 53859941 | rs368658799 | p.Ile97Val | Benign | missense_variant | 1 | 251476 | 3,97652E-06 |
| 53859948 | rs1342557197 | p.Ile99Thr | Pathogenic | missense_variant | 1 | 251476 | 3,97652E-06 |
| 53859960 | rs1203776934 | p.Gly103Asp | Pathogenic | missense_variant | 1 | 251474 | 3,97655E-06 |
| 53859976 | rs1253634102 | p.Tyr108Ter | Pathogenic | stop_gained | 1 | 251462 | 3,97674E-06 |
| 53859977 | rs769204154 | p.Leu109Val | Pathogenic | missense_variant | 1 | 251462 | 3,97674E-06 |
| 53859984 | rs1191932724 | p.Thr111Asn | Pathogenic | missense_variant | 1 | 251454 | 3,97687E-06 |
| 53859987 | rs1392454854 | p.Arg112Thr | Pathogenic | missense_variant | 1 | 251440 | 3,97709E-06 |
| 53860001 | rs759168981 | p.Pro117Ser | Pathogenic | missense_variant | 1 | 251444 | 3,97703E-06 |
| 53860016 | rs867050060 | p.Gly122Trp | Pathogenic | missense_variant | 1 | 31402 | 3,18451E-05 |
| 53860039 | rs757157849 | p.Glu129Asp | Benign | missense_variant | 1 | 251416 | 3,97747E-06 |
| 53860096 | rs141915969 | p.Ile148Met | Benign | missense_variant | 1 | 251428 | 3,97728E-06 |
| 53860100 | rs775311728 | p.Thr150Ala | Benign | missense_variant | 1 | 251434 | 3,97719E-06 |
| 53860104 | rs1322199273 | p.Ile151Thr | Benign | missense_variant | 1 | 251446 | 3,977E-06 |
| 53860105 | rs762625200 | p.Ile151Met | Benign | missense_variant | 1 | 251432 | 3,97722E-06 |
| 53860118 | rs751353294 | p.Glu156Lys | Benign | missense_variant | 1 | 251442 | 3,97706E-06 |
| 53860122 | rs1400616279 | p.Leu157Pro | Pathogenic | missense_variant | 1 | 31402 | 3,18451E-05 |
| 53860137 | rs1292567537 | p.Lys162Arg | Benign | missense_variant | 1 | 251454 | 3,97687E-06 |
| 53860142 | rs1231516982 | p.Asn164His | Benign | missense_variant | 1 | 251456 | 3,97684E-06 |
| 53860144 | rs1332447878 | p.Asn164Lys | Benign | missense_variant | 1 | 31400 | 3,18471E-05 |
| 53860157 | rs756242925 | p.Pro169Thr | Pathogenic | missense_variant | 1 | 251464 | 3,97671E-06 |
| 53860157 | rs756242925 | p.Pro169Ser | Benign | missense_variant | 1 | 251464 | 3,97671E-06 |
| 53860179 | rs779309219 | p.Phe176Cys | Benign | missense_variant | 1 | 251468 | 3,97665E-06 |
| 53860184 | rs1407999347 | p.Arg178Gly | Benign | missense_variant | 1 | 251470 | 3,97662E-06 |
| 53860222 | rs1282396852 | p.Glu190Asp | Benign | missense_variant | 1 | 251474 | 3,97655E-06 |
| 53860229 | rs1212033225 | p.Ile193Val | Benign | missense_variant | 1 | 251476 | 3,97652E-06 |
| 53860233 | rs1257170214 | p.Lys194Arg | Benign | missense_variant | 1 | 251478 | 3,97649E-06 |
| 53860241 | rs774050560 | p.Ala197Thr | Benign | missense_variant | 1 | 251460 | 3,97678E-06 |
| 53860244 | rs761651552 | p.Ala198Thr | Benign | missense_variant | 1 | 251460 | 3,97678E-06 |
| 53860262 | rs760541418 | p.Leu204Met | Pathogenic | missense_variant | 1 | 251420 | 3,97741E-06 |
| 53860270 | rs753902912 | p.Phe206Leu | Benign | missense_variant | 1 | 251374 | 3,97814E-06 |
| 53860271 | rs138348216 | p.Met207Leu | Pathogenic | missense_variant | 1 | 251368 | 3,97823E-06 |
| 53860281 | rs779165603 | p.Gln210Arg | Benign | missense_variant | 1 | 251284 | 3,97956E-06 |
| 53860298 | rs1028237381 | p.Lys216Gln | Pathogenic | missense_variant | 1 | 250988 | 3,98425E-06 |
| 53860300 | rs370009039 | p.Lys216Asn | Pathogenic | missense_variant | 1 | 250970 | 3,98454E-06 |
| 53860334 | rs1179039850 | p.Val228Leu | Pathogenic | missense_variant | 1 | 249806 | 4,00311E-06 |
| 53860344 | rs771907956 | p.His231Arg | Pathogenic | missense_variant | 1 | 249582 | 4,0067E-06 |
| 53860362 | rs1192563467 | p.Val237Ala | Pathogenic | missense_variant | 1 | 249450 | 4,00882E-06 |
| 53860371 | rs760665708 | p.Ser240Ter | Pathogenic | stop_gained | 1 | 249330 | 4,01075E-06 |
| 53860374 | rs753958759 | p.Ala241Val | Benign | missense_variant | 1 | 249302 | 4,0112E-06 |
| 53860390 | rs1302917355 | p.Ser246Arg | Pathogenic | missense_variant | 1 | 249224 | 4,01245E-06 |
| 53878070 | rs776580855 | p.Pro252Arg | Pathogenic | missense_variant | 1 | 251302 | 3,97928E-06 |
| 53878094 | rs76762929 | p.Ser260Cys | Benign | missense_variant | 1 | 251338 | 3,97871E-06 |
| 53878096 | rs1486647785 | p.His261Tyr | Benign | missense_variant | 1 | 251356 | 3,97842E-06 |
| 53878114 | rs1016337772 | p.Pro267Thr | Benign | missense_variant | 1 | 251380 | 3,97804E-06 |
| 53878115 | rs1475617180 | p.Pro267His | Benign | missense_variant | 1 | 251378 | 3,97807E-06 |
| 53878157 | rs1380871124 | p.Glu281Val | Benign | missense_variant | 1 | 251388 | 3,97791E-06 |
| 53878171 | rs376957531 | p.Ala286Thr | Benign | missense_variant | 1 | 251370 | 3,9782E-06 |
| 53878177 | rs777382917 | p.Pro288Thr | Pathogenic | missense_variant | 1 | 251370 | 3,9782E-06 |
| 53878186 | rs1223419681 | p.Gln291Ter | Benign | stop_gained | 1 | 31374 | 3,18735E-05 |
| 53878199 | rs1259762053 | p.Tyr295Cys | Pathogenic | missense_variant | 1 | 251344 | 3,97861E-06 |
| 53878210 | rs777558537 | p.Asp299His | Pathogenic | missense_variant | 1 | 251196 | 3,98096E-06 |
| 53907712 | rs766767212 | p.Thr304Ala | Pathogenic | missense_variant | 1 | 251386 | 3,97795E-06 |
| 53907724 | rs542365655 | p.Cys308Arg | Pathogenic | missense_variant | 1 | 251390 | 3,97788E-06 |
| 53907736 |  | p.Gly312Ser | Pathogenic | missense_variant | 1 | 251328 | 3,97886E-06 |
| 53907740 | rs1462889039 | p.Ser313Leu | Benign | missense_variant | 1 | 251342 | 3,97864E-06 |
| 53907742 | rs139814987 | p.Gln314Lys | Benign | missense_variant | 1 | 251324 | 3,97893E-06 |
| 53907749 | rs121918214 | p.Arg316Gln | Pathogenic | missense_variant | 1 | 251264 | 3,97988E-06 |
| 53907751 | rs1370044478 | p.Phe317Ile | Pathogenic | missense_variant | 1 | 251280 | 3,97962E-06 |
| 53907758 | rs781028867 | p.Ser319Tyr | Pathogenic | missense_variant | 1 | 251220 | 3,98057E-06 |
| 53907766 | rs200201735 | p.Arg322Gly | Pathogenic | missense_variant | 1 | 251148 | 3,98172E-06 |
| 53913756 | rs779204175 | p.Cys326Arg | Pathogenic | missense_variant | 1 | 251376 | 3,9781E-06 |
| 53913758 | rs1287663418 | p.Cys326Cys | Benign | splice_region_variant | 1 | 31358 | 3,18898E-05 |
| 53913769 | rs773329068 | p.Thr330Ile | Pathogenic | missense_variant | 1 | 251366 | 3,97826E-06 |
| 53913802 | rs1284248706 | p.Ala341Asp | Pathogenic | missense_variant | 1 | 251422 | 3,97738E-06 |
| 53913829 | rs377073096 | p.Asp350Gly | Benign | missense_variant | 1 | 251402 | 3,97769E-06 |
| 53913832 | rs1178634936 | p.Asn351Ser | Benign | missense_variant | 1 | 251414 | 3,9775E-06 |
| 53913880 | rs1435299421 | p.Gly367Glu | Benign | missense_variant | 1 | 251272 | 3,97975E-06 |
| 53922771 | rs747080793 | p.Trp383Arg | Pathogenic | missense_variant | 1 | 251412 | 3,97753E-06 |
| 53922778 | rs757468268 | p.Gln385Pro | Pathogenic | missense_variant | 1 | 251392 | 3,97785E-06 |
| 53922783 | rs781495764 | p.Asn387Asp | Benign | missense_variant | 1 | 251400 | 3,97772E-06 |
| 53922787 | rs780342015 | p.Arg388Gln | Pathogenic | missense_variant | 1 | 251384 | 3,97798E-06 |
| 53922798 | rs1465157191 | p.Cys392Arg | Pathogenic | missense_variant | 1 | 251390 | 3,97788E-06 |
| 53922805 | rs1441842690 | p.Asp394Gly | Pathogenic | missense_variant | 1 | 251408 | 3,9776E-06 |
| 53922812 | rs201572715 | p.Trp396Ter | Pathogenic | stop_gained | 1 | 251428 | 3,97728E-06 |
| 53922825 | rs1271268742 | p.Ala401Thr | Benign | missense_variant | 1 | 251430 | 3,97725E-06 |
| 53945301 | rs1205507227 | p.Arg4Gly | Unknown | missense_variant | 1 | 137586 | 7,26818E-06 |
| 53945304 | rs925727658 | p.Lys5Gln | Unknown | missense_variant | 1 | 31402 | 3,18451E-05 |
| 53945313 | rs1189932504 | p.Glu8Lys | Benign | missense_variant | 1 | 137508 | 7,2723E-06 |
| 53945320 | rs75097003 | p.Asn10Ser | Unknown | missense_variant | 1 | 137478 | 7,27389E-06 |
| 53945326 | rs1366935034 | p.Val12Ala | Unknown | missense_variant | 1 | 137424 | 7,27675E-06 |
| 53945328 | rs1008775419 | p.Glu13Lys | Pathogenic | missense_variant | 1 | 137392 | 7,27844E-06 |
| 53945328 | rs1008775419 | p.Glu13Ter | Pathogenic | stop_gained | 1 | 31388 | 3,18593E-05 |
| 53945361 | rs1424620579 | p.Arg24Cys | Unknown | missense_variant | 1 | 31396 | 3,18512E-05 |
| 53945362 | rs755788528 | p.Arg24Leu | Unknown | missense_variant | 1 | 137298 | 7,28343E-06 |
| 53945370 | rs1280940657 | p.His27Tyr | Unknown | missense_variant | 1 | 137296 | 7,28353E-06 |
| 53945374 | rs777553242 | p.His28Pro | Unknown | missense_variant | 1 | 137284 | 7,28417E-06 |
| 53945374 | rs777553242 | p.His28Leu | Unknown | missense_variant | 1 | 137284 | 7,28417E-06 |
| 53945381 | rs992422634 | p.Lys30Asn | Unknown | missense_variant | 1 | 137260 | 7,28544E-06 |
| 53945385 | rs1421133978 | p.Phe32Val | Unknown | missense_variant | 1 | 137242 | 7,2864E-06 |
| 53945396 | rs1380126914 | p.Met35Ile | Unknown | missense_variant | 1 | 137216 | 7,28778E-06 |
| 53967916 | rs1222652247 | p.Glu420Gly | Benign | missense_variant | 1 | 31382 | 3,18654E-05 |
| 53967918 | rs748820946 | p.Val421Ile | Benign | missense_variant | 1 | 251292 | 3,97943E-06 |
| 53967950 | rs1488347423 | p.Arg431Ser | Benign | missense_variant | 1 | 251370 | 3,9782E-06 |
| 53967969 | rs1192322094 | p.Ile438Val | Benign | missense_variant | 1 | 251386 | 3,97795E-06 |
| 53967981 |  | p.Leu442Phe | Pathogenic | missense_variant | 1 | 251384 | 3,97798E-06 |
| 53968008 | rs1399267613 | p.Glu451Gln | Pathogenic | missense_variant | 1 | 251346 | 3,97858E-06 |
| 53968019 | rs747478505 | p.Ala454Ala | Benign | splice_region_variant | 1 | 251330 | 3,97883E-06 |
| 53968021 | rs755686716 | p.Arg455Met | Pathogenic | missense_variant | 1 | 251304 | 3,97924E-06 |
| 54018858 | rs1450294366 | p.Arg30Met | Unknown | missense_variant | 1 | 134512 | 7,43428E-06 |
| 54018866 | rs1284787398 | p.Gly33Arg | Unknown | missense_variant | 1 | 134492 | 7,43539E-06 |
| 54145677 | rs1333596595 | p.Ala34Val | Unknown | missense_variant | 1 | 251104 | 3,98241E-06 |
| 54145694 | rs779678337 | p.Glu40Ter | Unknown | stop_gained | 1 | 251400 | 3,97772E-06 |
| 54145701 | rs1277348916 | p.Leu464Phe | Pathogenic | missense_variant | 1 | 251440 | 3,97709E-06 |
| 54145708 | rs747801335 | p.Asp467Asn | Pathogenic | missense_variant | 1 | 251452 | 3,9769E-06 |
| 54145708 | rs747801335 | p.Asp467Tyr | Pathogenic | missense_variant | 1 | 251452 | 3,9769E-06 |
| 54145724 | rs1291247246 | p.Cys472Tyr | Benign | missense_variant | 1 | 31398 | 3,18492E-05 |
| 54145726 | rs1254596324 | p.Arg473Trp | Benign | missense_variant | 1 | 251474 | 3,97655E-06 |
| 54145743 | rs1412828427 | p.Lys478Asn | Benign | missense_variant | 1 | 251492 | 3,97627E-06 |
| 54145759 | rs1329766069 | p.Pro484Ser | Pathogenic | missense_variant | 1 | 251486 | 3,97636E-06 |
| 54145766 | rs776819552 | p.Pro486Leu | Pathogenic | missense_variant | 1 | 31402 | 3,18451E-05 |
| 54145771 | rs1487911815 | p.Asp488Asn | Benign | missense_variant | 1 | 31406 | 3,1841E-05 |
| 54145774 | rs370137051 | p.Leu489Val | Pathogenic | missense_variant | 1 | 251486 | 3,97636E-06 |
| 54145796 | rs750897231 | p.Leu496Pro | Pathogenic | missense_variant | 1 | 251490 | 3,9763E-06 |
| 54145812 | rs1421215513 | p.Trp79Ter | Unknown | stop_gained | 1 | 31394 | 3,18532E-05 |
| 54145813 | rs1476316498 | p.Glu502Ter | Benign | stop_gained | 1 | 31380 | 3,18674E-05 |
| 54145813 | rs1476316498 | p.Glu502Gln | Benign | missense_variant | 1 | 251482 | 3,97643E-06 |
| 54145824 | rs1424370756 | p.Pro83Arg | Unknown | missense_variant | 1 | 251460 | 3,97678E-06 |
| 54145841 | rs1393406068 | p.Leu89Phe | Unknown | missense_variant | 1 | 251384 | 3,97798E-06 |
| 54145851 | rs770709958 | p.Arg92Met | Unknown | missense_variant | 1 | 251218 | 3,98061E-06 |
| 54145857 | rs1216468868 | p.Lys94Arg | Unknown | missense_variant | 1 | 251066 | 3,98302E-06 |
| 54145859 | rs781021746 | p.Arg95Ter | Unknown | stop_gained | 1 | 250968 | 3,98457E-06 |
| 54145887 | rs1388976674 | p.Val104Ala | Unknown | missense_variant | 1 | 249156 | 4,01355E-06 |
| 54145920 | rs757006993 | p.Ser115Phe | Unknown | missense_variant | 1 | 240790 | 4,153E-06 |
| 54145925 | rs1240856803 | p.Pro117Ser | Pathogenic | missense_variant | 1 | 237728 | 4,20649E-06 |
| 54145931 | rs1203482144 | p.Asp119Asn | Unknown | missense_variant | 1 | 235342 | 4,24914E-06 |
| 54145937 | rs745847276 | p.Ser121Gly | Unknown | missense_variant | 1 | 231016 | 4,3287E-06 |
| 54145944 | rs1159857501 | p.Arg123Gln | Unknown | missense_variant | 1 | 224034 | 4,46361E-06 |
| 54145949 | rs748318601 | p.Pro125Ser | Unknown | missense_variant | 1 | 220108 | 4,54322E-06 |

^a^ Five predictors: FATHMM, PANTHER, SIFT, PROVEAN and POLYPHEN-2.
