# Supplementary material for: Analysis of coding variants in the human FTO gene from the gnomAD database
Source: PLoS One. 2022 Jan 6;17(1):e0248610. doi: 10.1371/journal.pone.0248610 (PMC8735611; doi:10.1371/journal.pone.0248610)
Supplement: S3 Table — (DOCX) [file pone.0248610.s003.docx]

S3 Table. Missense variants in the FTO gene found in the gnomAD database and pathogenicity based on five predictor programs.

| **SUBSTITUTION** | **FATHMM** | **PANTHER** | **SIFT** | **PROVEAN** | **POLYPHEN-2** | **PREDICTION** |
| --- | --- | --- | --- | --- | --- | --- |
| p.Lys2Glu | Tolerated | Probably Damaging | Damaging | Neutral | Probably Damaging | Pathogenic |
| p.Lys2Asn | Damaging | Probably Damaging | Damaging | Neutral | Probably Damaging | Pathogenic |
| p.Arg3Cys | Damaging | Possibly Damaging | Damaging | Deleterious | Probably Damaging | Pathogenic |
| p.Arg3Pro | Damaging | Possibly Damaging | Damaging | Neutral | Probably Damaging | Pathogenic |
| p.Thr4Pro | Tolerated | Probably Benign | Tolerated | Neutral | Benign | Benign |
| p.Thr4Ala | Tolerated | Probably Benign | Tolerated | Neutral | Benign | Benign |
| p.Thr4Ile | Tolerated | Probably Benign | Tolerated | Neutral | Benign | Benign |
| p.Pro5Leu | Tolerated | Probably Benign | Tolerated | Neutral | Benign | Benign |
| p.Thr6Ala | Tolerated | Probably Benign | Tolerated | Neutral | Benign | Benign |
| p.Thr6Ile | Tolerated | Probably Benign | Damaging | Neutral | Possibly Damaging | Benign |
| p.Glu8Lys | Tolerated | Probably Benign | Damaging | Neutral | Possibly Damaging | Benign |
| p.Glu8Gly | Tolerated | Probably Benign | Damaging | Neutral | Benign | Benign |
| p.Arg10Ter | Tolerated | Probably Benign | Damaging | Neutral | Benign | Benign |
| p.Arg10Leu | Tolerated | Probably Benign | Damaging | Neutral | Benign | Benign |
| p.Glu11Asp | Tolerated | Possibly Damaging | Damaging | Neutral | Probably Damaging | Pathogenic |
| p.Ala14Thr | Tolerated | Possibly Damaging | Damaging | Neutral | Probably Damaging | Pathogenic |
| p.Lys16Gln | Tolerated | Possibly Damaging | Damaging | Neutral | Probably Damaging | Pathogenic |
| p.Lys16Asn | Damaging | Possibly Damaging | Damaging | Neutral | Probably Damaging | Pathogenic |
| p.Asp25Gly | Tolerated | Probably Benign | Damaging | Deleterious | Possibly Damaging | Pathogenic |
| p.Thr26Ala | Tolerated | Probably Benign | Tolerated | Neutral | Benign | Benign |
| p.Thr26Ile | Tolerated | Probably Benign | Damaging | Neutral | Possibly Damaging | Benign |
| p.Tyr30His | Damaging | Probably Damaging | Damaging | Deleterious | Probably Damaging | Pathogenic |
| p.Thr32Ser | Tolerated | Possibly Damaging | Tolerated | Neutral | Possibly Damaging | Benign |
| p.Thr32Ala | Tolerated | Possibly Damaging | Damaging | Neutral | Possibly Damaging | Pathogenic |
| p.Thr32Ile | Damaging | Possibly Damaging | Damaging | Deleterious | Probably Damaging | Pathogenic |
| p.Pro33Thr | Damaging | Probably Damaging | Damaging | Deleterious | Probably Damaging | Pathogenic |
| p.Asp36Glu | Tolerated | Probably Benign | Tolerated | Neutral | Possibly Damaging | Benign |
| p.Glu37Ter | Tolerated | Probably Benign | Tolerated | Neutral | Possibly Damaging | Benign |
| p.Phe38Leu | Damaging | Probably Damaging | Damaging | Deleterious | Probably Damaging | Pathogenic |
| p.Tyr39Cys | Damaging | Probably Benign | Tolerated | Deleterious | Probably Damaging | Pathogenic |
| p.Trp42Ter | Tolerated | Probably Damaging | Tolerated | Deleterious | Probably Damaging | Pathogenic |
| p.Gln43His | Tolerated | Probably Benign | Tolerated | Neutral | Benign | Benign |
| p.Pro47Ser | Tolerated | Probably Benign | Tolerated | Neutral | Benign | Benign |
| p.Lys48Gln | Tolerated | Possibly Damaging | Tolerated | Neutral | Probably Damaging | Benign |
| p.Ile50Leu | Tolerated | Possibly Damaging | Tolerated | Neutral | Benign | Benign |
| p.Leu51Phe | Tolerated | Probably Damaging | Tolerated | Neutral | Benign | Benign |
| p.Leu51Ile | Tolerated | Probably Damaging | Tolerated | Neutral | Possibly Damaging | Benign |
| p.Arg52Gln | Tolerated | Possibly Damaging | Tolerated | Neutral | Probably Damaging | Benign |
| p.Ser56Arg | Tolerated | Possibly Damaging | Tolerated | Neutral | Benign | Benign |
| p.Ser58Tyr | Damaging | Probably Benign | Damaging | Neutral | Possibly Damaging | Pathogenic |
| p.His62Arg | Damaging | Probably Damaging | Damaging | Deleterious | Probably Damaging | Pathogenic |
| p.Glu64Gly | Tolerated | Probably Benign | Tolerated | Deleterious | Probably Damaging | Benign |
| p.Val65Ile | Tolerated | Probably Damaging | Tolerated | Neutral | Probably Damaging | Benign |
| p.Val65Phe | Tolerated | Probably Damaging | Damaging | Deleterious | Probably Damaging | Pathogenic |
| p.Ala68Gly | Damaging | Probably Damaging | Tolerated | Neutral | Probably Damaging | Pathogenic |
| p.Phe69Leu | Tolerated | Probably Damaging | Tolerated | Deleterious | Probably Damaging | Pathogenic |
| p.Leu70Phe | Damaging | Probably Damaging | Damaging | Neutral | Benign | Pathogenic |
| p.Thr71Ile | Tolerated | Possibly Damaging | Damaging | Deleterious | Benign | Pathogenic |
| p.His73Arg | Tolerated | Probably Benign | Tolerated | Neutral | Benign | Benign |
| p.His75Gln | Tolerated | Possibly Damaging | Damaging | Neutral | Probably Damaging | Pathogenic |
| p.Gly76Asp | Tolerated | Probably Damaging | Damaging | Deleterious | Probably Damaging | Pathogenic |
| p.Gly76Val | Damaging | Probably Damaging | Damaging | Deleterious | Probably Damaging | Pathogenic |
| p.Cys77Arg | Tolerated | Probably Damaging | Damaging | Deleterious | Probably Damaging | Pathogenic |
| p.Arg80Trp | Damaging | Probably Benign | Damaging | Deleterious | Probably Damaging | Pathogenic |
| p.Asp81Gly | Damaging | Probably Damaging | Damaging | Deleterious | Probably Damaging | Pathogenic |
| p.Leu82Pro | Damaging | Probably Damaging | Damaging | Deleterious | Probably Damaging | Pathogenic |
| p.Val83Ile | Tolerated | Probably Damaging | Tolerated | Neutral | Probably Damaging | Benign |
| p.Val83Leu | Tolerated | Probably Damaging | Tolerated | Neutral | Probably Damaging | Benign |
| p.Val83Phe | Damaging | Probably Damaging | Damaging | Deleterious | Probably Damaging | Pathogenic |
| p.Arg84Ser | Tolerated | Probably Damaging | Tolerated | Deleterious | Probably Damaging | Pathogenic |
| p.Ile85Phe | Tolerated | Possibly Damaging | Tolerated | Neutral | Probably Damaging | Benign |
| p.Gln86Ter | Tolerated | Probably Benign | Tolerated | Neutral | Possibly Damaging | Benign |
| p.Gln86Pro | Tolerated | Probably Benign | Tolerated | Neutral | Possibly Damaging | Benign |
| p.Lys88Arg | Tolerated | Possibly Damaging | Tolerated | Neutral | Probably Damaging | Benign |
| p.Pro93Leu | Tolerated | Probably Damaging | Tolerated | Deleterious | Probably Damaging | Pathogenic |
| p.Pro93Arg | Tolerated | Probably Damaging | Tolerated | Deleterious | Probably Damaging | Pathogenic |
| p.Arg96Cys | Damaging | Probably Damaging | Damaging | Deleterious | Probably Damaging | Pathogenic |
| p.Arg96Pro | Damaging | Probably Damaging | Damaging | Deleterious | Probably Damaging | Pathogenic |
| p.Arg96His | Damaging | Probably Damaging | Damaging | Deleterious | Probably Damaging | Pathogenic |
| p.Ile97Val | Tolerated | Probably Benign | Tolerated | Neutral | Benign | Benign |
| p.Ile99Val | Tolerated | Possibly Damaging | Tolerated | Neutral | Benign | Benign |
| p.Ile99Thr | Damaging | Possibly Damaging | Damaging | Deleterious | Probably Damaging | Pathogenic |
| p.Gly103Asp | Damaging | Probably Damaging | Damaging | Deleterious | Probably Damaging | Pathogenic |
| p.Tyr106His | Damaging | Probably Damaging | Damaging | Deleterious | Probably Damaging | Pathogenic |
| p.Tyr108Ter | Damaging | Probably Damaging | Damaging | Deleterious | Probably Damaging | Pathogenic |
| p.Leu109Val | Damaging | Probably Damaging | Damaging | Neutral | Probably Damaging | Pathogenic |
| p.Thr111Asn | Damaging | Probably Damaging | Tolerated | Deleterious | Probably Damaging | Pathogenic |
| p.Arg112Thr | Damaging | Probably Damaging | Damaging | Deleterious | Probably Damaging | Pathogenic |
| p.Thr115Met | Damaging | Possibly Damaging | Damaging | Deleterious | Probably Damaging | Pathogenic |
| p.Pro117Ser | Damaging | Probably Damaging | Damaging | Deleterious | Probably Damaging | Pathogenic |
| p.Val120Met | Tolerated | Probably Benign | Tolerated | Neutral | Benign | Benign |
| p.Gly122Trp | Damaging | Possibly Damaging | Damaging | Deleterious | Probably Damaging | Pathogenic |
| p.Lys126Arg | Tolerated | Possibly Damaging | Damaging | Neutral | Benign | Benign |
| p.Glu129Lys | Tolerated | Probably Benign | Tolerated | Neutral | Benign | Benign |
| p.Glu129Asp | Tolerated | Probably Benign | Tolerated | Neutral | Benign | Benign |
| p.Ala130Pro | Tolerated | Probably Benign | Tolerated | Neutral | Benign | Benign |
| p.Ile132Thr | Tolerated | Possibly Damaging | Tolerated | Neutral | Benign | Benign |
| p.Ala134Thr | Tolerated | Probably Benign | Tolerated | Neutral | Benign | Benign |
| p.Asn143Ser | Damaging | Probably Damaging | Damaging | Deleterious | Probably Damaging | Pathogenic |
| p.Asp144Asn | Tolerated | Possibly Damaging | Tolerated | Neutral | Possibly Damaging | Benign |
| p.Leu146Met | Damaging | Possibly Damaging | Tolerated | Neutral | Probably Damaging | Pathogenic |
| p.Ile148Met | Tolerated | Probably Benign | Tolerated | Neutral | Benign | Benign |
| p.Thr150Ala | Tolerated | Probably Damaging | Tolerated | Neutral | Probably Damaging | Benign |
| p.Ile151Thr | Tolerated | Probably Benign | Tolerated | Neutral | Benign | Benign |
| p.Ile151Met | Tolerated | Probably Benign | Tolerated | Neutral | Possibly Damaging | Benign |
| p.Glu156Lys | Tolerated | Probably Benign | Tolerated | Neutral | Benign | Benign |
| p.Leu157Pro | Damaging | Possibly Damaging | Tolerated | Neutral | Possibly Damaging | Pathogenic |
| p.Lys162Arg | Tolerated | Probably Benign | Tolerated | Neutral | Benign | Benign |
| p.Ala163Thr | Tolerated | Probably Benign | Tolerated | Neutral | Benign | Benign |
| p.Asn164His | Tolerated | Probably Benign | Damaging | Neutral | Possibly Damaging | Benign |
| p.Asn164Lys | Tolerated | Probably Benign | Tolerated | Neutral | Benign | Benign |
| p.Pro169Thr | Tolerated | Possibly Damaging | Damaging | Neutral | Probably Damaging | Pathogenic |
| p.Pro169Ser | Tolerated | Possibly Damaging | Tolerated | Neutral | Probably Damaging | Benign |
| p.Leu170Trp | Damaging | Probably Benign | Tolerated | Neutral | Possibly Damaging | Benign |
| p.Phe176Ile | Tolerated | Probably Benign | Tolerated | Neutral | Possibly Damaging | Benign |
| p.Phe176Cys | Tolerated | Probably Benign | Damaging | Neutral | Probably Damaging | Benign |
| p.Arg178Gly | Tolerated | Probably Benign | Tolerated | Neutral | Benign | Benign |
| p.Gly182Ala | Tolerated | Probably Benign | Tolerated | Neutral | Possibly Damaging | Benign |
| p.Ser184Pro | Tolerated | Probably Benign | Tolerated | Neutral | Probably Damaging | Benign |
| p.Tyr185Cys | Tolerated | Probably Benign | Tolerated | Neutral | Benign | Benign |
| p.Gly187Arg | Tolerated | Probably Benign | Tolerated | Neutral | Benign | Benign |
| p.Glu190Lys | Tolerated | Probably Benign | Tolerated | Neutral | Possibly Damaging | Benign |
| p.Glu190Asp | Tolerated | Probably Benign | Tolerated | Neutral | Benign | Benign |
| p.Ile193Val | Tolerated | Probably Benign | Tolerated | Neutral | Benign | Benign |
| p.Lys194Arg | Tolerated | Possibly Damaging | Tolerated | Neutral | Benign | Benign |
| p.Ala197Thr | Tolerated | Probably Benign | Tolerated | Neutral | Benign | Benign |
| p.Ala198Thr | Tolerated | Probably Benign | Tolerated | Neutral | Benign | Benign |
| p.Val201Ile | Tolerated | Probably Benign | Damaging | Neutral | Possibly Damaging | Benign |
| p.Leu204Met | Damaging | Possibly Damaging | Damaging | Neutral | Probably Damaging | Pathogenic |
| p.Phe206Leu | Tolerated | Probably Benign | Tolerated | Neutral | Possibly Damaging | Benign |
| p.Met207Val | Tolerated | Probably Damaging | Damaging | Deleterious | Probably Damaging | Pathogenic |
| p.Met207Leu | Tolerated | Probably Damaging | Damaging | Deleterious | Possibly Damaging | Pathogenic |
| p.Gln210Arg | Tolerated | Possibly Damaging | Tolerated | Neutral | Benign | Benign |
| p.Gln210His | Tolerated | Possibly Damaging | Tolerated | Neutral | Possibly Damaging | Benign |
| p.Lys216Gln | Damaging | Probably Damaging | Damaging | Deleterious | Probably Damaging | Pathogenic |
| p.Lys216Asn | Damaging | Probably Damaging | Damaging | Deleterious | Probably Damaging | Pathogenic |
| p.Tyr220Cys | Damaging | Probably Damaging | Damaging | Deleterious | Probably Damaging | Pathogenic |
| p.Met223Val | Tolerated | Probably Damaging | Damaging | Deleterious | Probably Damaging | Pathogenic |
| p.Met223Ile | Tolerated | Probably Damaging | Damaging | Deleterious | Probably Damaging | Pathogenic |
| p.Ala227Ser | Tolerated | Probably Damaging | Tolerated | Neutral | Probably Damaging | Benign |
| p.Val228Leu | Damaging | Probably Damaging | Damaging | Deleterious | Probably Damaging | Pathogenic |
| p.His231Arg | Damaging | Probably Damaging | Damaging | Deleterious | Probably Damaging | Pathogenic |
| p.Val237Ala | Tolerated | Probably Damaging | Damaging | Neutral | Probably Damaging | Pathogenic |
| p.Ser240Ter | Damaging | Probably Damaging | Damaging | Deleterious | Probably Damaging | Pathogenic |
| p.Ala241Thr | Tolerated | Probably Benign | Tolerated | Neutral | Benign | Benign |
| p.Ala241Val | Tolerated | Probably Benign | Damaging | Neutral | Possibly Damaging | Benign |
| p.Ser246Arg | Tolerated | Possibly Damaging | Damaging | Neutral | Possibly Damaging | Pathogenic |
| p.Gly251Asp | Tolerated | Probably Damaging | Tolerated | Neutral | Benign | Benign |
| p.Gly251Gly | Synonymous | Probably Damaging | Tolerated | Neutral | Synonymous | Benign |
| p.Pro252Arg | Tolerated | Possibly Damaging | Damaging | Neutral | Possibly Damaging | Pathogenic |
| p.Glu253Lys | Tolerated | Possibly Damaging | Tolerated | Neutral | Benign | Benign |
| p.Ser256Asn | Tolerated | Probably Benign | Tolerated | Neutral | Possibly Damaging | Benign |
| p.Asp258Gly | Tolerated | Probably Benign | Tolerated | Neutral | Possibly Damaging | Benign |
| p.Ser260Cys | Damaging | Probably Benign | Tolerated | Neutral | Possibly Damaging | Benign |
| p.Ser260Tyr | Tolerated | Probably Benign | Tolerated | Neutral | Possibly Damaging | Benign |
| p.His261Tyr | Tolerated | Probably Benign | Damaging | Neutral | Benign | Benign |
| p.Leu262Pro | Tolerated | Probably Benign | Damaging | Neutral | Benign | Benign |
| p.Glu263Lys | Tolerated | Possibly Damaging | Tolerated | Neutral | Probably Damaging | Benign |
| p.Gly264Asp | Tolerated | Probably Damaging | Tolerated | Neutral | Possibly Damaging | Benign |
| p.Arg265Gly | Tolerated | Possibly Damaging | Damaging | Neutral | Probably Damaging | Pathogenic |
| p.Pro267Thr | Tolerated | Probably Damaging | Tolerated | Neutral | Probably Damaging | Benign |
| p.Pro267His | Tolerated | Probably Damaging | Tolerated | Neutral | Probably Damaging | Benign |
| p.Asp268Gly | Tolerated | Probably Benign | Tolerated | Neutral | Possibly Damaging | Benign |
| p.His271Arg | Tolerated | Possibly Damaging | Tolerated | Neutral | Probably Damaging | Benign |
| p.Val272Ala | Tolerated | Probably Damaging | Damaging | Neutral | Probably Damaging | Pathogenic |
| p.Glu281Val | Tolerated | Probably Benign | Tolerated | Neutral | Probably Damaging | Benign |
| p.Glu281Asp | Tolerated | Probably Benign | Tolerated | Neutral | Benign | Benign |
| p.Ala286Thr | Tolerated | Possibly Damaging | Tolerated | Neutral | Benign | Benign |
| p.Ala286Val | Tolerated | Possibly Damaging | Tolerated | Neutral | Possibly Damaging | Benign |
| p.Ile287Thr | Tolerated | Probably Benign | Tolerated | Neutral | Possibly Damaging | Benign |
| p.Pro288Thr | Damaging | Probably Damaging | Damaging | Deleterious | Probably Damaging | Pathogenic |
| p.Gln291Ter | Tolerated | Possibly Damaging | Tolerated | Neutral | Benign | Benign |
| p.Asp293Val | Damaging | Probably Damaging | Damaging | Deleterious | Probably Damaging | Pathogenic |
| p.Tyr295Cys | Damaging | Probably Damaging | Damaging | Deleterious | Probably Damaging | Pathogenic |
| p.Leu298Phe | Tolerated | Possibly Damaging | Damaging | Neutral | Probably Damaging | Pathogenic |
| p.Asp299His | Damaging | Probably Damaging | Damaging | Deleterious | Probably Damaging | Pathogenic |
| p.Thr304Ala | Tolerated | Probably Damaging | Damaging | Deleterious | Possibly Damaging | Pathogenic |
| p.Gln306Lys | Tolerated | Probably Damaging | Damaging | Neutral | Probably Damaging | Pathogenic |
| p.Cys308Arg | Damaging | Probably Damaging | Damaging | Deleterious | Probably Damaging | Pathogenic |
| p.Gly312Ser | Damaging | Probably Damaging | Damaging | Deleterious | Probably Damaging | Pathogenic |
| p.Ser313Leu | Tolerated | Probably Benign | Tolerated | Neutral | Benign | Benign |
| p.Gln314Lys | Tolerated | Probably Benign | Tolerated | Neutral | Benign | Benign |
| p.Arg316Trp | Damaging | Probably Damaging | Damaging | Deleterious | Possibly Damaging | Pathogenic |
| p.Arg316Gln | Damaging | Probably Damaging | Damaging | Deleterious | Probably Damaging | Pathogenic |
| p.Phe317Ile | Damaging | Probably Damaging | Damaging | Deleterious | Probably Damaging | Pathogenic |
| p.Ser319Tyr | Damaging | Probably Damaging | Damaging | Deleterious | Probably Damaging | Pathogenic |
| p.Arg322Gly | Damaging | Probably Damaging | Damaging | Deleterious | Probably Damaging | Pathogenic |
| p.Arg322Ter | Damaging | Probably Damaging | Damaging | Deleterious | Probably Damaging | Pathogenic |
| p.Arg322Gln | Damaging | Probably Damaging | Damaging | Deleterious | Probably Damaging | Pathogenic |
| p.Glu325Val | Tolerated | Possibly Damaging | Tolerated | Deleterious | Probably Damaging | Pathogenic |
| p.Glu325Glu | Synonymous | Possibly Damaging | Tolerated | Neutral | Synonymous | Benign |
| p.Cys326Ser | Tolerated | Possibly Damaging | Tolerated | Deleterious | Benign | Benign |
| p.Cys326Arg | Tolerated | Possibly Damaging | Damaging | Deleterious | Probably Damaging | Pathogenic |
| p.Cys326Cys | Synonymous | Possibly Damaging | Tolerated | Neutral | Synonymous | Benign |
| p.Thr330Ile | Damaging | Probably Damaging | Damaging | Deleterious | Possibly Damaging | Pathogenic |
| p.Asp332Gly | Tolerated | Probably Benign | Damaging | Deleterious | Possibly Damaging | Pathogenic |
| p.Tyr333Cys | Damaging | Probably Damaging | Damaging | Deleterious | Probably Damaging | Pathogenic |
| p.Gln336Pro | Tolerated | Probably Benign | Tolerated | Neutral | Possibly Damaging | Benign |
| p.Arg337Cys | Damaging | Probably Benign | Damaging | Deleterious | Probably Damaging | Pathogenic |
| p.Arg337His | Damaging | Probably Benign | Damaging | Deleterious | Probably Damaging | Pathogenic |
| p.Cys338Arg | Damaging | Probably Damaging | Damaging | Deleterious | Probably Damaging | Pathogenic |
| p.Gln339His | Tolerated | Probably Benign | Tolerated | Neutral | Benign | Benign |
| p.Ala341Ser | Damaging | Probably Damaging | Damaging | Deleterious | Probably Damaging | Pathogenic |
| p.Ala341Thr | Damaging | Probably Damaging | Damaging | Deleterious | Possibly Damaging | Pathogenic |
| p.Ala341Asp | Damaging | Probably Damaging | Damaging | Deleterious | Probably Damaging | Pathogenic |
| p.Asn344Asp | Tolerated | Probably Damaging | Damaging | Neutral | Probably Damaging | Pathogenic |
| p.Asp348Val | Tolerated | Probably Benign | Tolerated | Deleterious | Benign | Benign |
| p.Asp350Gly | Tolerated | Probably Benign | Damaging | Neutral | Possibly Damaging | Benign |
| p.Asn351Ser | Tolerated | Probably Benign | Tolerated | Neutral | Benign | Benign |
| p.Ser358Phe | Damaging | Probably Damaging | Damaging | Deleterious | Probably Damaging | Pathogenic |
| p.Pro361Ser | Tolerated | Probably Damaging | Tolerated | Neutral | Benign | Benign |
| p.Lys365Arg | Tolerated | Possibly Damaging | Tolerated | Neutral | Possibly Damaging | Benign |
| p.Gly367Glu | Tolerated | Possibly Damaging | Tolerated | Neutral | Probably Damaging | Benign |
| p.Glu373Val | Damaging | Probably Damaging | Damaging | Deleterious | Probably Damaging | Pathogenic |
| p.Val374Val | Synonymous | Probably Damaging | Tolerated | Neutral | Synonymous | Benign |
| p.Glu375Lys | Damaging | Probably Damaging | Damaging | Deleterious | Probably Damaging | Pathogenic |
| p.Trp383Arg | Damaging | Probably Damaging | Damaging | Deleterious | Probably Damaging | Pathogenic |
| p.Gln385Pro | Damaging | Probably Damaging | Damaging | Deleterious | Probably Damaging | Pathogenic |
| p.Asn387Asp | Tolerated | Probably Benign | Tolerated | Neutral | Benign | Benign |
| p.Arg388Ter | Damaging | Probably Damaging | Tolerated | Deleterious | Probably Damaging | Pathogenic |
| p.Arg388Gln | Damaging | Probably Damaging | Tolerated | Neutral | Probably Damaging | Pathogenic |
| p.Arg388Pro | Damaging | Probably Damaging | Damaging | Deleterious | Probably Damaging | Pathogenic |
| p.Cys392Arg | Tolerated | Probably Damaging | Tolerated | Deleterious | Probably Damaging | Pathogenic |
| p.Thr393Ile | Tolerated | Possibly Damaging | Damaging | Deleterious | Probably Damaging | Pathogenic |
| p.Asp394Gly | Tolerated | Possibly Damaging | Damaging | Neutral | Probably Damaging | Pathogenic |
| p.Trp396Ter | Damaging | Probably Damaging | Damaging | Deleterious | Probably Damaging | Pathogenic |
| p.Pro399Ala | Tolerated | Probably Damaging | Tolerated | Deleterious | Possibly Damaging | Pathogenic |
| p.Pro399Ser | Tolerated | Probably Damaging | Tolerated | Deleterious | Probably Damaging | Pathogenic |
| p.Ala401Thr | Tolerated | Probably Benign | Tolerated | Neutral | Benign | Benign |
| p.Gln402His | Tolerated | Probably Benign | Tolerated | Neutral | Benign | Benign |
| p.Ala405Val | Tolerated | Probably Benign | Tolerated | Neutral | Benign | Benign |
| p.Arg4Gly | Unknown | Unknown | Unknown | Unknown | Unknown | Unknown |
| p.Lys5Gln | Unknown | Unknown | Unknown | Unknown | Unknown | Unknown |
| p.Val6Ala | Unknown | Unknown | Unknown | Unknown | Unknown | Unknown |
| p.Glu8Lys | Tolerated | Probably Benign | Damaging | Neutral | Possibly Damaging | Benign |
| p.Cys9Tyr | Unknown | Unknown | Unknown | Unknown | Unknown | Unknown |
| p.Asn10Ser | Unknown | Unknown | Unknown | Unknown | Unknown | Unknown |
| p.Ser11Thr | Unknown | Unknown | Unknown | Unknown | Unknown | Unknown |
| p.Val12Ala | Unknown | Unknown | Unknown | Unknown | Unknown | Unknown |
| p.Glu13Lys | Tolerated | Possibly Damaging | Damaging | Neutral | Probably Damaging | Pathogenic |
| p.Glu13Ter | Tolerated | Possibly Damaging | Damaging | Neutral | Probably Damaging | Pathogenic |
| p.Pro14Ser | Unknown | Unknown | Unknown | Unknown | Unknown | Unknown |
| p.Cys15Ter | Unknown | Unknown | Unknown | Unknown | Unknown | Unknown |
| p.Lys20Glu | Unknown | Unknown | Unknown | Unknown | Unknown | Unknown |
| p.Tyr23Cys | Unknown | Unknown | Unknown | Unknown | Unknown | Unknown |
| p.Arg24Cys | Unknown | Unknown | Unknown | Unknown | Unknown | Unknown |
| p.Arg24Leu | Unknown | Unknown | Unknown | Unknown | Unknown | Unknown |
| p.Arg24His | Unknown | Unknown | Unknown | Unknown | Unknown | Unknown |
| p.Cys25Arg | Unknown | Unknown | Unknown | Unknown | Unknown | Unknown |
| p.Ile26Val | Unknown | Unknown | Unknown | Unknown | Unknown | Unknown |
| p.His27Tyr | Unknown | Unknown | Unknown | Unknown | Unknown | Unknown |
| p.His28Pro | Unknown | Unknown | Unknown | Unknown | Unknown | Unknown |
| p.His28Arg | Unknown | Unknown | Unknown | Unknown | Unknown | Unknown |
| p.His28Leu | Unknown | Unknown | Unknown | Unknown | Unknown | Unknown |
| p.Gly29Arg | Unknown | Unknown | Unknown | Unknown | Unknown | Unknown |
| p.Lys30Asn | Unknown | Unknown | Unknown | Unknown | Unknown | Unknown |
| p.Phe32Val | Unknown | Unknown | Unknown | Unknown | Unknown | Unknown |
| p.Met35Ile | Unknown | Unknown | Unknown | Unknown | Unknown | Unknown |
| p.His419Arg | Tolerated | Probably Benign | Tolerated | Neutral | Benign | Benign |
| p.Glu420Gly | Tolerated | Probably Benign | Tolerated | Neutral | Possibly Damaging | Benign |
| p.Val421Phe | Tolerated | Probably Benign | Tolerated | Neutral | Possibly Damaging | Benign |
| p.Val421Ile | Tolerated | Probably Benign | Tolerated | Neutral | Benign | Benign |
| p.Gly425Val | Tolerated | Probably Benign | Tolerated | Neutral | Benign | Benign |
| p.Leu426Phe | Tolerated | Probably Benign | Tolerated | Neutral | Possibly Damaging | Benign |
| p.Val428Met | Tolerated | Probably Benign | Tolerated | Neutral | Benign | Benign |
| p.Arg431Ser | Tolerated | Probably Benign | Tolerated | Neutral | Benign | Benign |
| p.Asn432Ser | Tolerated | Possibly Damaging | Tolerated | Neutral | Benign | Benign |
| p.Ile438Val | Tolerated | Probably Benign | Tolerated | Neutral | Benign | Benign |
| p.Leu439Arg | Damaging | Probably Damaging | Damaging | Deleterious | Probably Damaging | Pathogenic |
| p.Ser441Leu | Tolerated | Probably Benign | Tolerated | Neutral | Benign | Benign |
| p.Leu442Phe | Damaging | Probably Damaging | Damaging | Neutral | Probably Damaging | Pathogenic |
| p.Arg445Cys | Damaging | Probably Damaging | Damaging | Deleterious | Probably Damaging | Pathogenic |
| p.Arg445His | Damaging | Probably Damaging | Damaging | Deleterious | Probably Damaging | Pathogenic |
| p.Arg449Trp | Damaging | Probably Damaging | Damaging | Deleterious | Probably Damaging | Pathogenic |
| p.Glu451Gln | Tolerated | Possibly Damaging | Damaging | Neutral | Probably Damaging | Pathogenic |
| p.Ala454Ala | Synonymous | Probably Benign | Tolerated | Neutral | Synonymous | Benign |
| p.Arg455Lys | Damaging | Probably Damaging | Damaging | Neutral | Probably Damaging | Pathogenic |
| p.Arg455Met | Damaging | Probably Damaging | Damaging | Deleterious | Probably Damaging | Pathogenic |
| p.Arg30Met | Unknown | Unknown | Unknown | Unknown | Unknown | Unknown |
| p.Gly33Arg | Unknown | Unknown | Unknown | Unknown | Unknown | Unknown |
| p.Arg455Ser | Damaging | Probably Damaging | Damaging | Deleterious | Probably Damaging | Pathogenic |
| p.Ala34Val | Unknown | Unknown | Unknown | Unknown | Unknown | Unknown |
| p.Arg459Ter | Tolerated | Probably Benign | Tolerated | Neutral | Probably Damaging | Benign |
| p.Arg459Gln | Tolerated | Probably Benign | Tolerated | Neutral | Possibly Damaging | Benign |
| p.Arg462Ter | Tolerated | Probably Benign | Tolerated | Neutral | Possibly Damaging | Benign |
| p.Arg462Gln | Tolerated | Probably Benign | Tolerated | Neutral | Benign | Benign |
| p.Glu40Ter | Unknown | Unknown | Unknown | Unknown | Unknown | Unknown |
| p.Leu464Phe | Damaging | Probably Damaging | Damaging | Neutral | Probably Damaging | Pathogenic |
| p.Pro465Ser | Damaging | Probably Damaging | Damaging | Deleterious | Benign | Pathogenic |
| p.Pro465His | Damaging | Probably Damaging | Damaging | Deleterious | Probably Damaging | Pathogenic |
| p.Pro465Leu | Damaging | Probably Damaging | Damaging | Deleterious | Probably Damaging | Pathogenic |
| p.Asp467Asn | Tolerated | Possibly Damaging | Damaging | Neutral | Possibly Damaging | Pathogenic |
| p.Asp467Tyr | Damaging | Possibly Damaging | Damaging | Deleterious | Possibly Damaging | Pathogenic |
| p.Glu471Gly | Tolerated | Probably Benign | Tolerated | Neutral | Benign | Benign |
| p.Cys472Tyr | Tolerated | Probably Damaging | Tolerated | Neutral | Probably Damaging | Benign |
| p.Arg473Trp | Damaging | Probably Benign | Tolerated | Neutral | Benign | Benign |
| p.Arg473Gln | Tolerated | Probably Benign | Tolerated | Neutral | Probably Damaging | Benign |
| p.Lys478Glu | Tolerated | Probably Benign | Tolerated | Neutral | Benign | Benign |
| p.Lys478Asn | Tolerated | Probably Benign | Tolerated | Neutral | Benign | Benign |
| p.Asp480Asn | Tolerated | Probably Benign | Tolerated | Neutral | Possibly Damaging | Benign |
| p.Arg60Ter | Unknown | Unknown | Unknown | Unknown | Unknown | Unknown |
| p.Arg60Gln | Unknown | Unknown | Unknown | Unknown | Unknown | Unknown |
| p.Pro484Ser | Damaging | Probably Damaging | Damaging | Deleterious | Probably Damaging | Pathogenic |
| p.Pro486Leu | Damaging | Possibly Damaging | Damaging | Deleterious | Probably Damaging | Pathogenic |
| p.Arg64His | Unknown | Unknown | Unknown | Unknown | Unknown | Unknown |
| p.Asp488Asn | Tolerated | Probably Benign | Tolerated | Neutral | Benign | Benign |
| p.Leu489Val | Damaging | Probably Damaging | Damaging | Neutral | Probably Damaging | Pathogenic |
| p.Leu489Phe | Damaging | Probably Damaging | Damaging | Deleterious | Probably Damaging | Pathogenic |
| p.Ile492Val | Tolerated | Probably Benign | Tolerated | Neutral | Benign | Benign |
| p.Ile492Thr | Damaging | Probably Benign | Tolerated | Neutral | Benign | Benign |
| p.Ser70Leu | Unknown | Unknown | Unknown | Unknown | Unknown | Unknown |
| p.Val493Phe | Tolerated | Probably Benign | Damaging | Neutral | Possibly Damaging | Benign |
| p.Val493Ile | Tolerated | Probably Benign | Tolerated | Neutral | Benign | Benign |
| p.Gln72Arg | Unknown | Unknown | Unknown | Unknown | Unknown | Unknown |
| p.Asn73Ser | Unknown | Unknown | Unknown | Unknown | Unknown | Unknown |
| p.Leu496His | Damaging | Probably Benign | Damaging | Deleterious | Probably Damaging | Pathogenic |
| p.Leu496Pro | Damaging | Probably Benign | Damaging | Deleterious | Probably Damaging | Pathogenic |
| p.Trp79Ter | Unknown | Unknown | Unknown | Unknown | Unknown | Unknown |
| p.Glu502Ter | Tolerated | Probably Benign | Damaging | Neutral | Benign | Benign |
| p.Glu502Gln | Tolerated | Probably Benign | Damaging | Neutral | Benign | Benign |
| p.Pro505Thr | Tolerated | Probably Benign | Damaging | Neutral | Benign | Benign |
| p.Pro83Arg | Unknown | Unknown | Unknown | Unknown | Unknown | Unknown |
| p.Ser86Arg | Unknown | Unknown | Unknown | Unknown | Unknown | Unknown |
| p.Leu89Phe | Unknown | Unknown | Unknown | Unknown | Unknown | Unknown |
| p.Arg91Trp | Unknown | Unknown | Unknown | Unknown | Unknown | Unknown |
| p.Arg91Gln | Unknown | Unknown | Unknown | Unknown | Unknown | Unknown |
| p.Arg92Lys | Unknown | Unknown | Unknown | Unknown | Unknown | Unknown |
| p.Arg92Met | Unknown | Unknown | Unknown | Unknown | Unknown | Unknown |
| p.Lys94Arg | Unknown | Unknown | Unknown | Unknown | Unknown | Unknown |
| p.Arg95Ter | Unknown | Unknown | Unknown | Unknown | Unknown | Unknown |
| p.Arg95Gly | Unknown | Unknown | Unknown | Unknown | Unknown | Unknown |
| p.Arg97Trp | Unknown | Unknown | Unknown | Unknown | Unknown | Unknown |
| p.Arg97Gln | Unknown | Unknown | Unknown | Unknown | Unknown | Unknown |
| p.Leu98Ile | Damaging | Probably Damaging | Damaging | Neutral | Probably Damaging | Pathogenic |
| p.Phe99Ser | Unknown | Unknown | Unknown | Unknown | Unknown | Unknown |
| p.Ser100Phe | Unknown | Unknown | Unknown | Unknown | Unknown | Unknown |
| p.Asn102Ser | Unknown | Unknown | Unknown | Unknown | Unknown | Unknown |
| p.Val103Phe | Unknown | Unknown | Unknown | Unknown | Unknown | Unknown |
| p.Val103Ile | Unknown | Unknown | Unknown | Unknown | Unknown | Unknown |
| p.Val104Ala | Unknown | Unknown | Unknown | Unknown | Unknown | Unknown |
| p.Ser110Thr | Unknown | Unknown | Unknown | Unknown | Unknown | Unknown |
| p.Asp113Glu | Unknown | Unknown | Unknown | Unknown | Unknown | Unknown |
| p.Ser115Phe | Unknown | Unknown | Unknown | Unknown | Unknown | Unknown |
| p.Pro117Ser | Damaging | Probably Damaging | Damaging | Deleterious | Probably Damaging | Pathogenic |
| p.Leu118Ile | Unknown | Unknown | Unknown | Unknown | Unknown | Unknown |
| p.Asp119Asn | Unknown | Unknown | Unknown | Unknown | Unknown | Unknown |
| p.Cys120Tyr | Unknown | Unknown | Unknown | Unknown | Unknown | Unknown |
| p.Ser121Gly | Unknown | Unknown | Unknown | Unknown | Unknown | Unknown |
| p.Thr122Ser | Unknown | Unknown | Unknown | Unknown | Unknown | Unknown |
| p.Thr122Ile | Unknown | Unknown | Unknown | Unknown | Unknown | Unknown |
| p.Arg123Trp | Unknown | Unknown | Unknown | Unknown | Unknown | Unknown |
| p.Arg123Gln | Unknown | Unknown | Unknown | Unknown | Unknown | Unknown |
| p.Pro125Ser | Unknown | Unknown | Unknown | Unknown | Unknown | Unknown |
| p.Ile126Val | Unknown | Unknown | Unknown | Unknown | Unknown | Unknown |
| p.Ser129Asn | Unknown | Unknown | Unknown | Unknown | Unknown | Unknown |
